# Coumarin/nitrogen-bearing heterocyclic hybrid-loaded electrospun PMMA/PVP nanofibrous scaffolds for accelerating topical wound healing rates: synthesis and *in vitro* bio-evaluation

**DOI:** 10.1039/d5ra02535d

**Published:** 2025-10-03

**Authors:** Samar A. Salim, Mohamed A. M. Ali, Tasneem Abed, Anis Ahmad Chaudhary, Fehmi Boufahja, Asmaa Mohammed Hasanein, Eman Abdelaziz, Shahira H. EL-Moslamy, Amr Negm, Ibrahim E. T. El Sayed, Elbadawy A. Kamoun, Mohamed A. Hawata

**Affiliations:** a Nanotechnology Research Center (NTRC), The British University in Egypt El-Sherouk City Cairo 11837 Egypt; b Department of Biology, College of Science, Imam Mohammad Ibn Saud Islamic University (IMSIU) Riyadh 11623 Saudi Arabia; c Badr University in Cairo Research Center, Badr University in Cairo Badr City Cairo 11829 Egypt; d Department of Chemistry, Faculty of Science, Menoufia University Shibin El Kom 32511 Egypt ibrahimtantawy@yahoo.co.uk; e Bioprocess Development Department (BID), Genetic Engineering and Biotechnology Research Institute (GEBRI), City of Scientific Research and Technological Applications (SRTA-City) New Borg El-Arab City Alexandria 21934 Egypt; f Department of Chemistry, College of Science, King Faisal University Al-Ahsa 31982 Saudi Arabia anegm@kfu.edu.sa ekamoun@kfu.edu.sa +201283320302

## Abstract

Coumarin-nitrogen heterocyclic compounds (*e.g.* quinoline, acridine, and phthalazine) were synthesized by facile reactions and elucidated by FTIR, ^1^H, and ^13^C-NMR analyses, which showed consistency with the expected structures. Coumarin-quinoline (drug A)- and coumarin-acridine (drug B)-loaded electrospun PMMA/PVP nanofibrous scaffolds were fabricated using electrospinning techniques. Results showed a successful PMMA/PVP blend, which formed the matrix that was used as the main scaffold for drug loading/release. The drugs (A and B) were encapsulated within the matrix as verified by IR and SEM results. Bio-evaluation through cytotoxicity and anticancer screening was conducted using the MTT assay against lung fibroblast (Wi-38), colon carcinoma (Caco-2), lung carcinoma (A549) and breast carcinoma (MDA) cell lines. Notably, the IC_50_ values of the synthesized derivatives against Wi-38 cells were found in the range of 126.3–195.0 μg mL^−1^, indicating a high level of safety for the synthesized compounds toward the treated human normal (Wi-38) cell line. The IC_50_ values of these potent derivatives against Caco-2 cells were estimated in the range of 4.87–38.23 μg mL^−1^, with SI values ranging from 4.59 to 25.93. Their IC_50_ values against A549 cells were estimated to be in the range of 5.74–32.05 μg mL^−1^, with SI values ranging from 5.47 to 18.09. However, their IC_50_ values against MDA cells were estimated to be in the range of 4.27–14.91 μg mL^−1^, with SI values ranging from 11.76 to 29.58 μg mL^−1^. The antimicrobial activities of the two synthesized compounds toward Gram-positive compared to Gram-negative bacteria were estimated; the highest antimicrobial activity was achieved at inhibition zones of ∼19.5 ± 2.3 and 17.2 ± 1.3 mm toward *S. aureus* and *S. mutans*, respectively, followed by *S. typhimurium* (∼15.5 ± 1.1 mm). According to the obtained findings, coumarin-nitrogen heterocyclic compounds (quinoline, acridine)-loaded PMMA/PVP electrospun NFs can be regarded as good antimicrobial biomaterials for different biomedical applications, particularly for wound dressings.

## Abstract 2

Coumarin-quinoline and coumarin-acridine compounds were synthesized, loaded into PMMA/PVP nanofibers, and demonstrated potent anticancer and antimicrobial activity, suggesting promise for wound dressing applications.

## Graphical abstract

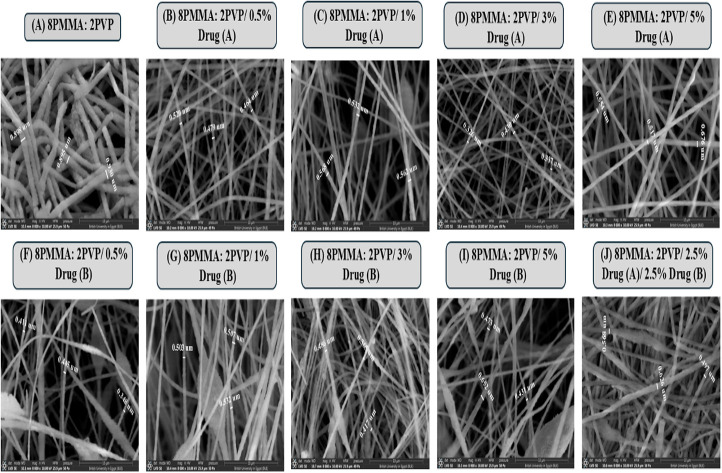

## 1. Introduction

Cancer and infectious diseases are among the most serious health problems faced by humanity today. Despite significant advances in treatment, both diseases continue to be major causes of death worldwide.^1^ In the search for new and more effective therapies, researchers have increasingly turned their attention to natural products and their derivatives. Among these, coumarin and nitrogen-containing heterocyclic compounds such as indoles, quinolines, and acridines, have emerged as promising candidates because of their miscellaneous biological activities.^2,3^ The development of effective therapeutic agents for combating cancer and microbial infections remains a critical challenge in medical science.^4^ Coumarin, found in several plants, has gained significant attention, owing to its varied pharmacological features, including anti-cancer,^5^ anti-inflammatory,^6^ and antimicrobial activities.^7^ The strategic modification and hybridization of coumarin derivatives can enhance their biological effectiveness and broaden their applicability in therapeutic contexts.^8^ Various current studies have stated the synthesis and biological activities of coumarin-containing nitrogen heterocyclic compound hybrids.^9–12^ Recently, the application of electrospun nanofibrous scaffolds has been developed as a promising path for delivering bioactive compounds.^13^ Among various polymer matrices, poly(methyl methacrylate) (PMMA) and polyvinyl pyrrolidone (PVP) stand out for their favorable mechanical properties, biocompatibility, and ability to facilitate the encapsulation of therapeutic agents.^14^ The electrospinning technique offers a versatile approach for generating nanofibrous structures with high surface area-to-volume ratios, enabling the controlled release of incorporated drugs while providing a supportive environment for cellular activities.^15^

Nanofibrous scaffolds have been widely employed in biomedical fields, owing to their remarkable characteristics, including a large surface area, high porosity, and the capability to mimic natural extracellular matrix (ECM).^16^ Among the various nanofiber production techniques, electrospinning has proven to be an adaptable method for generating nanofibrous scaffolds with controlled structure and functionality. Lately, the incorporation of bioactive compounds into these scaffolds has emerged as a promising strategy for improving their therapeutic potential. Limited recent studies have reported the use of electrospun nanofibrous platforms for drug delivery. For example, the arrangement of nanofibrous scaffolds loaded with the antimicrobial drug ciprofloxacin showed that the drug-loaded scaffolds exhibited the sustained release of ciprofloxacin and enhanced antimicrobial activity, compared to free ciprofloxacin.^17,18^

In this research, we present the synthesis and analysis of new hybrids featuring coumarin and nitrogen-containing heterocyclic compounds. The hybrids were then loaded onto electrospun PMMA/PVP nanofibrous scaffolds as a biomaterial carrier. The anticancer and antimicrobial activities of the hybrids and the drug-loaded scaffolds were assessed.

## 2. Materials and methods

### 2.1. Materials

The following materials were utilized: 4,7-dichloroquinoline (97%), triethylamine (99%), and coumarin-3-carboxylic acid (99%), all sourced from Sigma Aldrich, MO, USA. Other reagents, including 1,4-phenylenediamine, thionyl chloride (97%), and solvents such as ethanol (98%), dichloromethane (98%), and petroleum ether (60–80 °C), were provided by LOBA Chemie, Mumbai, India. Polyvinylpyrrolidone (PVP) (Mwt. 40 000 g mol^−1^) powder (C_6_H_9_NO)_*n*_ was obtained from Alfa Aesar, while polymethyl methacrylate (PMMA, Mwt. ~550 kDa) was acquired from Lucite International, UK. *N*,*N*-Dimethylformamide (DMF) was sourced from EMSURE VWR, Germany. Additionally, MTT (3-[4,5-dimethylthiazol-2-yl]-2,5-diphenyltetrazolium bromide) dye and dimethyl sulfoxide (DMSO, research grade) were bought from Serva Electrophoresis GmbH, Germany. The starting materials, such as 9-chloroacridine and 1-chloro-4-(*p*-tolyl)phthalazine, were prepared according to previously reported methods.^19–21^

Microbial strains: *Staphylococcus aureus* ATCC 25923 (*S. aureus*), *Streptococcus mutans* ATCC 25175 (*S. mutans*), *Bacillus cereus* ATCC 19637 (*B. cereus*), *Bacillus aureus* ATCC 25923 (*B. aureus*), *Bacillus epidermidis* ATCC 14990 (*B. epidermidis*), and *Bacillus subtilis* ATCC 11774 (*B. subtilis*) represent Gram-positive bacteria, whereas *Pseudomonas aeruginosa* ATCC 27853 (*P. aeruginosa*), *Klebsiella pneumonia* ATCC 13883 (*K. pneumonia*), and *Salmonella typhimurium* ATCC 14028 (*S. typhimurium*), *Salmonella pneumoniae* ATCC 10031(*S. pneumoniae*), *Escherichia coli* ATCC 10536 (*E. coli*), and *Salmonella paratyphi* ATCC 9150 (*S. paratyphi*) represent Gram-negative bacteria. Additionally, *Candida albicans* ATCC 10231 (*C. albicans*), *Candida krusei* ATCC 6258 (*C. krusei*), *Candida glabrata* ATCC 66032 (*C. glabrata*), and *Candida parapsilosis* ATCC 22019 (*C. parapsilosis*) were applied as models for unicellular fungi.

Cell lines: Human cancer cells, including Caco-2 (colon carcinoma), A549 (lung carcinoma), and MDA (breast carcinoma) cell lines, as compared to the normal human lung fibroblast (Wi-38) cells, were supplied by ATCC, USA. These tested human pathogens were taken from SRTA-City, Egypt.

### 2.2. Instrumental investigation

#### 2.2.1. Scanning electron microscopy (SEM) investigation

SEM analysis was conducted to assess the synthesized compounds and surface attributes of the combined electrospun nanofibers (NFs). Characterization was carried out using FE-SEM, model *Quattro S* from Thermo Scientific, USA. Samples were imaged without any coatings to provide a true representation of their features. To preserve the samples' integrity, a 5 kV accelerating voltage was used for evaluating the microstructure and morphology of electrospun fibers.

#### 2.2.2. FTIR analysis

FT-IR analysis was conducted to investigate the chemical configurations of the synthesized compounds and nanofiber (NF) scaffolds. FT-IR analyses were accomplished using an FT-IR instrument (model 8400s, Shimadzu, Japan). Spectral data were collected over a range of 4000–400 cm^−1^ to capture typical fingerprints and enhance the understanding of the molecular composition of scaffolds. Melting points (m.p.) were recorded on a scientific melting point apparatus, and are uncorrected.

### 2.3. Chemistry experimental

#### 2.3.1. Synthesis of coumarin-*N*-heterocyclic hybrids

Coumarin-3-carbonyl chloride (0.3 g, 1.27 mmol) and the appropriate amine (1.27 mmol) were dissolved in 2 mL of CH_2_Cl_2_. Triethylamine (0.386 g, 3.81 mmol) was then added dropwise while stirring at ambient conditions. The progress of the reaction was monitored using TLC until the starting materials were fully dispersed, which took three days. The reaction mixture was subsequently poured into ice-cold water and extracted three times with CH_2_Cl_2_. The organic layer was dried and filtered. The filtrate was then evaporated to remove the solvent, and the colored precipitate was filtered, dried, and recrystallized from ethanol, yielding a good amount of product.

#### 2.3.2. Synthesis of *N*-(4-((7-chloroquinolin-4-yl)amino)phenyl)-2-*oxo*-2*H*-chromene-3-carboxamide (the coumarin analog appended with quinoline) (drug A)

A brownish-yellow solid was obtained with a yield of 0.47 g (83%) and a melting point of 258–260 °C. FT-IR analysis (KBr) revealed characteristic peaks at 3390 cm^−1^ (NH), 1688 cm^−1^ (C–C=O), 1664 cm^−1^ (O–C=O), 1609 cm^−1^ (C=CAr), 1539 cm^−1^ (C=N), and 1203 cm^−1^ (C–C). The ^1^H-NMR spectrum displayed signals at *δ* ppm: 7.39–8.49 (m, 12H, CHAr), 8.53 (m, 1H, CH=C), 8.93 (s, 1H, CH=NAr), 9.62 (br.s, 1H, NH), and 10.73 (s, 1H, NH). Additionally, the ^13^C-NMR spectrum revealed chemical shifts at *δ* ppm: 116.44, 118.00, 118.63, 120.14, 124.17, 124.76, 125.03, 125.85, 126.58, 130.46, 134.87, 135.86, 136.01, 144.70, 147.50, 147.91, 149.40, 152.72, 154.04, and 160.62.

#### 2.3.3. Synthesis of *N*-(4-(acridin-9-ylamino) phenyl)-2-*oxo*-2*H*-chromene-3-carboxamide, (coumarin analog appended with acridine) (drug B)

An orange solid was obtained with a yield of 0.5 g (79.8%) and a melting point exceeding 300 °C. FT-IR analysis (KBr) showed characteristic peaks at 3231 cm^−1^ (NH), 1703 cm^−1^ (C–C=O), 1678 cm^−1^ (O–C=O), 1631 cm^−1^ (C=CAr), 1558 cm^−1^ (C=N), and 1221 cm^−1^ (C–C). ^1^H-NMR spectrum revealed signals at *δ* ppm: 5.7 (m, 2H, CH_2_), 7.03–7.80 (m, 15H, CHAr), 8.35 (m, 1H, CH=C), 8.92 (m, 1H, CH=NAr), 10.5 (s, 1H, NH), and 10.62 (br.s, 1H, NH). Additionally, the ^13^C-NMR spectrum displayed chemical shifts at *δ* ppm: 116.24, 118.02, 118.35, 118.72, 119.03, 120.10, 121.26, 122.94, 124.80, 124.85, 125.28, 125.47, 125.74, 126.50, 128.90, 130.13, 130.26, 132.48, 134.18, 134.46, 140.80, 147.08, 148.06, 153.32, and 153.84.

#### 2.3.4. Synthesis of 2-*oxo-N*-(4-((4-(*p*-tolyl)phthalazin-1-yl)amino)phenyl)-2H-chromene-3-carboxamide, (coumarin analog appended with acridine) (drug C)

A dark green solid was obtained with a yield of 0.45 g (75%) and a melting point of 260 °C. FT-IR analysis (KBr) indicated characteristic peaks at 3295 and 3053 cm^−1^ (NH), 1670 cm^−1^ (C–C=O), 1605 cm^−1^ (O–C=O), 1526 and 1510 cm^−1^ (C=N), and 1207 cm^−1^ (C–C). The ^1^H-NMR spectrum showed signals at *δ* ppm: 2.00 (m, 3H, CH3), 7.22–7.98 (m, 16H, CHAr), 8.58 (m, 1H, CH=NAr), 8.66 (m, 1H, CH=C), 9.65 (br.s, 1H, NH), and 10.66 (s, 1H, NH). Additionally, the ^13^C-NMR spectrum displayed chemical shifts at *δ* ppm: 124.91, 125.36, 126.06, 126.58, 126.62, 126.88, 126.95, 127.40, 127.90, 129.09, 129.20, 129.62, 129.77, 129.88, 130.28, 130.81, 131.56, 132.18, 132.52, 133.55, 133.66, 134.20, 134.36, 136.68, 138.41, and 139.37, with a peak at 146.39.

### 2.4. Fabrication of drug-loaded and unloaded electrospun PMMA/PVP nanofibrous scaffolds

The fabrication and spinning conditions optimization of the PMMA/PVP nanofibers (NFs) are discussed in detail. Two separate polymeric solutions of PVP (30%, w/v) and PMMA (30%, w/v) were prepared and dissolved in DMF. Both PVP and PMMA solutions were then merged in an 8 : 2, v/v volume ratio, resulting in a homogeneous PMMA/PVP solution. The combined solution was kept stirring overnight at 50 °C. Several solutions of different drug concentrations were added to the latter polymeric solution and kept stirring at ambient conditions for 2 hours, followed by sonication for 10 min to guarantee the mixing of drugs in the polymeric solution. NFs were made up from PMMA/PVP solutions, utilizing an electrospinner (MECC, NANON-01A, MECC, Japan) to regulate the optimal spinning settings. The regular mixtures were packed into a 5 mL syringe (22 G needle). Several electrospinning considerations were fixed as indicated in Table (S1) (SI), including the flow rate (0.5–1.0 mL h^−1^), voltage (22–27 kV), and fixed distance of 15 cm (tip-to-collector), width 40 mm, and speed 20 m s^−1^.

The fabrication of PMMA/PVP/drug (A) and PMMA/PVP/drug (B) NFs was conducted by adding drugs (A) and (B) separately with four concentrations for each drug (0.5, 1, 3, and 5%, w/v) to the optimum PMMA/PVP solution (8 : 2). Each solution was set by stirring the combination for 2 h at RT. For fabricating PVP/PMMA/drug (A)/drug (B) NFs, both drugs (A) and (B) were added to the optimal blended solution (8 : 2) at a concentration of 2.5% (w/v) for each drug. The electrospinning conditions were carried out as shown in Table S1 (SI), assembled at a width of ∼40 mm on a flat plate collector. All electrospinning trials were achieved in optimum conditions with a relative humidity of (H, 35%).

### 2.5. Bio-evaluation assays

#### 2.5.1. Cytotoxicity and anticancer activity assays

MTT assay: the normal cell line (Wi-38), colon cancer (Caco-2), lung cancer (A549), and breast cancer (MDA) cell lines were sourced from ATCC, USA. All different cell lines were cultivated and preserved in DMEM medium (SERANA, Germany), accompanied by 10% FBS (Gibco, USA) and 1% Pen/Strep solution (Lonza, USA). The newly prepared derivatives at various doses were considered for their anticancer activity and their safety on normal cells, applying MTT (3-[4, 5-dimethylthiazol]-2, 5-diphenyltetrazolium bromide) methods.^22^ At first, all normal and cancer cell lines (1.0 ×10^4^) were inoculated in 4 antiseptic 96-well tissue culture microplates and incubated for 24 h in a CO_2_ incubator. After cell attachments, the prepared derivatives were added to both normal Wi-38 cells at diverse concentrations of (6.25–200 μg mL^−1^, 2-fold) and cancer cells at different concentrations of (3.125–100 μg mL^−1^, 2-fold) in triplicate and incubated for another 48 h at 37 °C in a CO_2_ incubator with an adjusted concentration of 5%. After that, the treated cells were cleaned three times with PBS to remove debris and dead cells. Next, 200 μL of 0.5 mg mL^−1^ MTT dye solution in PBS was mixed in each well, and the cells were further kept for 3 h at 37 °C in a 5% CO_2_ incubator. After that, MTT was removed and 200 μL of DMSO was added to each well. The optical density (OD) was recorded at 570 nm *via* a microplate reader (B.M.G. LabTech, Germany). Basic cells (untreated) were utilized as negative control cells, and 5-fluorouracil (5-FU) was included as a standard chemotherapy drug. The qualified cell viability (%) was evaluated as in equation (eqn (1)):

1 Relative cell viability (%) = [*A*_1_ − (*A*_0_/*A*_u_) − *A*_0_] × 100

where *A*_1_ is the OD of the examined derivative, *A*_0_ is the OD of the standard, and *A*_U_ is the OD of the untreated cells.

Anticancer activity assay: the anticancer effect of each derivative was assessed by evaluating the half-maximal inhibitory concentration (IC_50_) value *via* the GraphPad Prism 7.0 software. However, SI (selective index) values of each derivative were estimated by splitting the IC_50_ value of healthy cells by the IC_50_ value of malignant cells, as previously reported.^23^ The effects of the created derivatives on the morphology of MDA cells were evaluated at IC_50_ doses and captured by phase-contrast microscopy (Olympus, Germany) in comparison to untreated control cells.

#### 2.5.2. Assaying of wound healing effect

To assess the anti-migration inhibition capability of newly synthesized derivatives, a wound healing technique was employed. A549 cells were planted in 12-well plates and kept alive at 37 °C until they achieved 80–90% fluency. The cell monolayers were smashed with a sterile tip and exposed to the prepared derivatives at IC_50_ doses. At 0 and 48 h of treatment, the wound closure area was captured and calculated by imaging analysis with the Cell-Sens software (Olympus, Japan). The reduction in all area closures was considered to determine the inhibition of migration (%) in treated cells *versus* untouched cells using the following equation (eqn (2)):

2 Migration inhibition rate (%) = 100 − (*A*_0_ − *A*_*t*_)/*A*_0_ × 100

where *A*_0_ is the scratch width at 0 h, and *A*_*t*_ is the scratch width at 48 h.

### 2.6. Antimicrobial activity

The antimicrobial activity of the prepared compounds was estimated through an agar-well diffusion method as adapted from the modified Kirby–Bauer protocol.^24^ In this test, microbial pathogens were applied, including *Staphylococcus aureus* ATCC 25923 (*S. aureus*), *Streptococcus mutans* ATCC 25175 (*S. mutans*), *Bacillus cereus* ATCC 19637 (*B. cereus*), *Bacillus aureus* ATCC 25923 (*B. aureus*), *Bacillus epidermidis* ATCC 14990 (*B. epidermidis*), and *Bacillus subtilis* ATCC 11774 (*B. subtilis*) as Gram-positive bacteria, and *Pseudomonas aeruginosa* ATCC 27853 (*P. aeruginosa*), *Klebsiella pneumonia* ATCC 13883 (*K. pneumonia*), and *Salmonella typhimurium* ATCC 14028 (*S. typhimurium*), *Salmonella pneumoniae* ATCC 10031(*S. pneumoniae*), *Escherichia coli* ATCC 10536 (*E. coli*), and *Salmonella paratyphi* ATCC 9150 (*S. paratyphi*) as Gram-negative bacteria. Additionally, *Candida albicans* (ATCC 10231), *Candida krusei* ATCC 6258 (*C. krusei*), *Candida glabrata* ATCC 66032 (*C. glabrata*), and*Candida parapsilosis* ATCC 22019 (*C. parapsilosis*) were applied as models of unicellular fungi. The pre-inoculation cultures of the pathogens were prepared on Muller–Hinton broth and cultivated for 24 h at 37 °C. The agar-well test was prepared on Muller–Hinton agar with 100 μL of each pathogen pre-culture (0.5 McFarland conc.) and uniformly distributed on the plate surface. Under aseptic conditions, six wells were created on the agar surface using a sterile corkborer (9 mm). To each well, 100 μL of the prepared compound (10 mg mL^−1^) was separately inserted into each plate. All plates were incubated for 24 h at 37 °C; the established inhibition zones (Halo-zones) surrounding the wells indicated positive antimicrobial activity and were stated as the radius of the inhibition zone (mm).

The antimicrobial qualities of formulations were evaluated in this study using a variety of techniques. The formulations including 8 PMMA and 2 PVP, which contained varying doses of drug A (0.5% = T1, 1% = T2, 3% = T3, and 5% = T4) or drug B (0.5% = T5, 1% = T6, 3% = T7, and 5% = T8), as well as a combination of 2.5% of drug A and 2.5% of drug B (T9) were assessed. The tested human pathogens were cultivated in a nutritional broth medium containing 0.5% peptone, 0.3% yeast extract, 0.2% beef extract, 0.1% glucose, and 0.5% NaCl in accordance with 0.5 McFarland turbidity criteria.^25^ To carry out the agar-well diffusion analysis, microbial cultures (100 μL) were spread on nutrient agar plates using sterile cotton swabs. After drilling each well with a sterile cork-borer of 5 mm in diameter, 50 μL of the tested formulations and the control (composed of 8 PMMA and 2 PVP) were loaded. Agar plates were thereafter left to incubate at 37 °C for an entire day. The inhibitory zone that surrounds each well was measured in millimeters (mm) using a ruler.^26^

Additionally, a spectrophotometric antibiofilm assay was utilized to determine whether the evaluated formulations might inhibit the growth of pathogens. Fresh nutritional broth was inoculated with each pathogen separately, and this mixture was then incubated at 37 °C at 200 rpm. The optical density (OD) at 600 nm was measured over a 6 h incubation period, allowing for an estimation of the starting microbe's exponential phase (3 × 10^7^ CFU mL^−1^). To produce treated cultures, 900 μL of planktonic cultures were collected aseptically with 100 μL of each formulation. Furthermore, untreated (formula-free) cultures of pathogens were grown as controls. The cultures were then incubated for 24 hours at 200 rpm and 37 °C. Through spectroscopic evaluation of microbial turbidity, the inhibitory effects of the examined formulations were determined.^27^ By comparing the OD of the treated sample with the matching controls, anti-biofilm percentages for each formula were computed.^28^

The formulations exhibiting the strongest antimicrobial activity against their respective pathogens were selected by time-kill kinetics experiments. Each microbial culture's inoculum (5 × 10^8^ CFU mL^−1^) was treated with the chosen formulation. Furthermore, control growth cultures were generated for every pathogen, devoid of the tested formulation. Evaluation was carried out on pathogen samples that had grown for 0, 6, 12, 18, 24, 30, 36, 42, and 48 hours at 200 rpm and 37 °C. After that, 100 μL of diluted samples were swabbed onto nutrient agar plates. After a 24 hour incubation period at 37 °C, the number of visible colonies was counted, and the CFU mL^−1^ was calculated.^29–31^ Pathogen cell biofilm reduction (%) in the treated samples relative to each control was then calculated using the logarithm of counted colonies (log_10_ CFU mL^−1^). The cell viability of treated and untreated samples at various time intervals was related by a stacked bar plot.^29^

Every test for the efficacy of antimicrobial treatments was carried out three times. The mean ± standard deviation (M ± SD) of the study was computed using Microsoft Excel 2019. The Minitab 18 program (MINITAB version 18.1) used Tukey's multiple comparison post hoc test to compute a one-way analysis of variance (ANOVA) and demonstrate statistical significance. A 95% confidence interval (*P*-value ≤ 0.05) was used to establish statistical significance.

## 3. Results and discussion

### 3.1. Chemistry experimental

#### 3.1.1. Synthesis of coumarin-3-carbonyl chloride

**Scheme 1:**
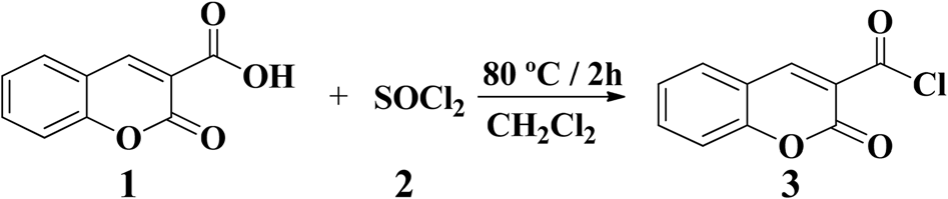
Synthesis of coumarin-3-carbonyl chloride (3).

The primary intermediate acid chloride 3 was successfully obtained in high yield as pale-yellow crystals, following previously reported procedures,^32,33^ starting from the commercially available coumarin-3-carboxylic acid 1 and thionyl chloride (SOCl_2_), as described in Scheme 1.

#### 3.1.2. Synthesis of diamino-substituted heterocycle hybrids

**Scheme 2:**
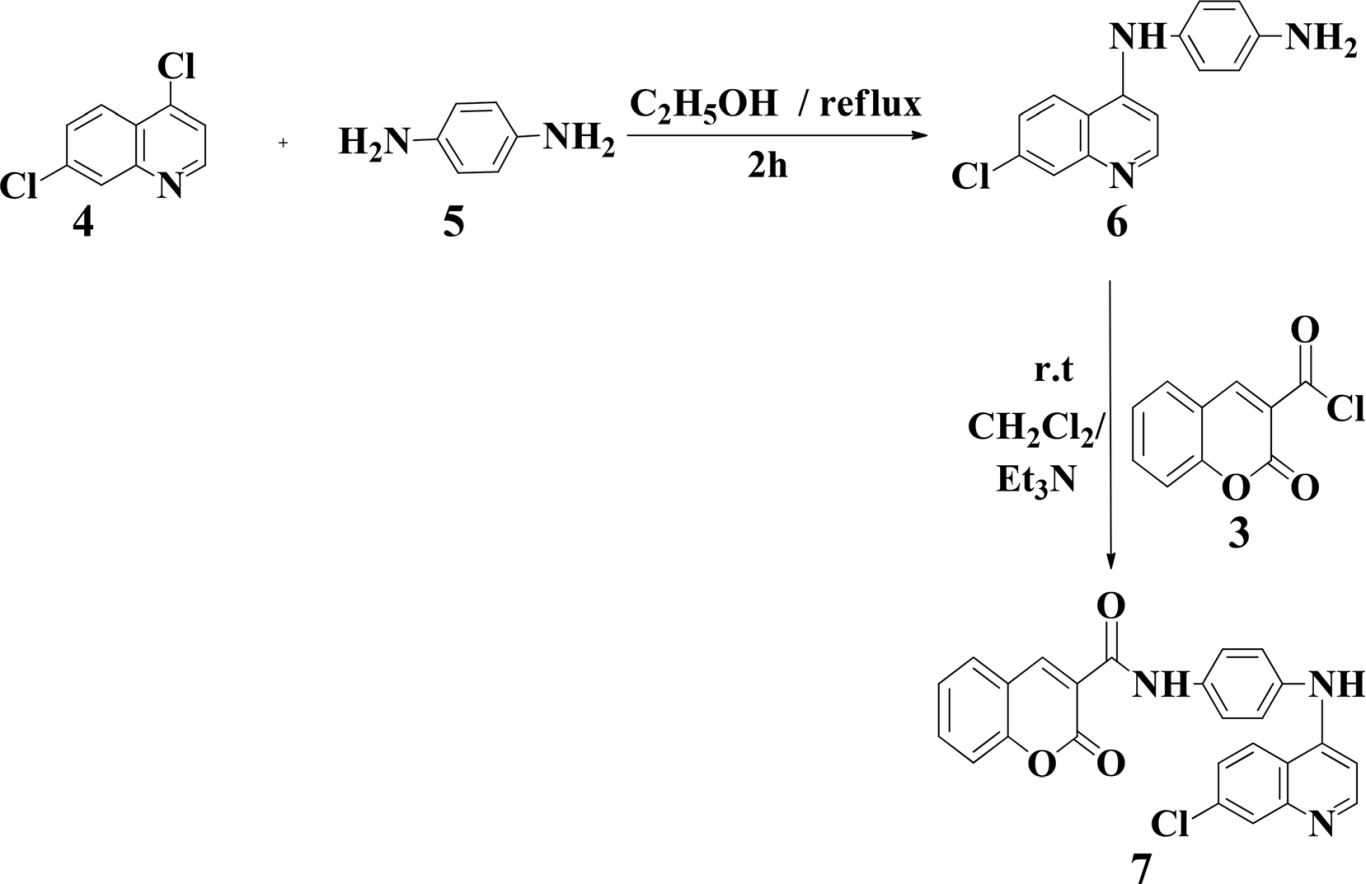
Production of coumarin-quinoline hybrid 7 (drug A).

The reaction of 4,7-dichloroquinoline 4 with aromatic diamine 5 in the presence of base catalyst afforded 4-bis-aminoquinoline 6 in great yield through nucleophilic aromatic substitution (*S*_NAr_), according to previous methods,^34,35^ as illustrated in Scheme 1. A synthetic method for the development of coumarin-quinoline hybrid 7 was accomplished by the reaction of 3 with diamine 6 in equimolar fractions in the presence of triethylamine as a base, presenting hybrid 7 in excellent yield (82%) Scheme 2.

**Scheme 3:**
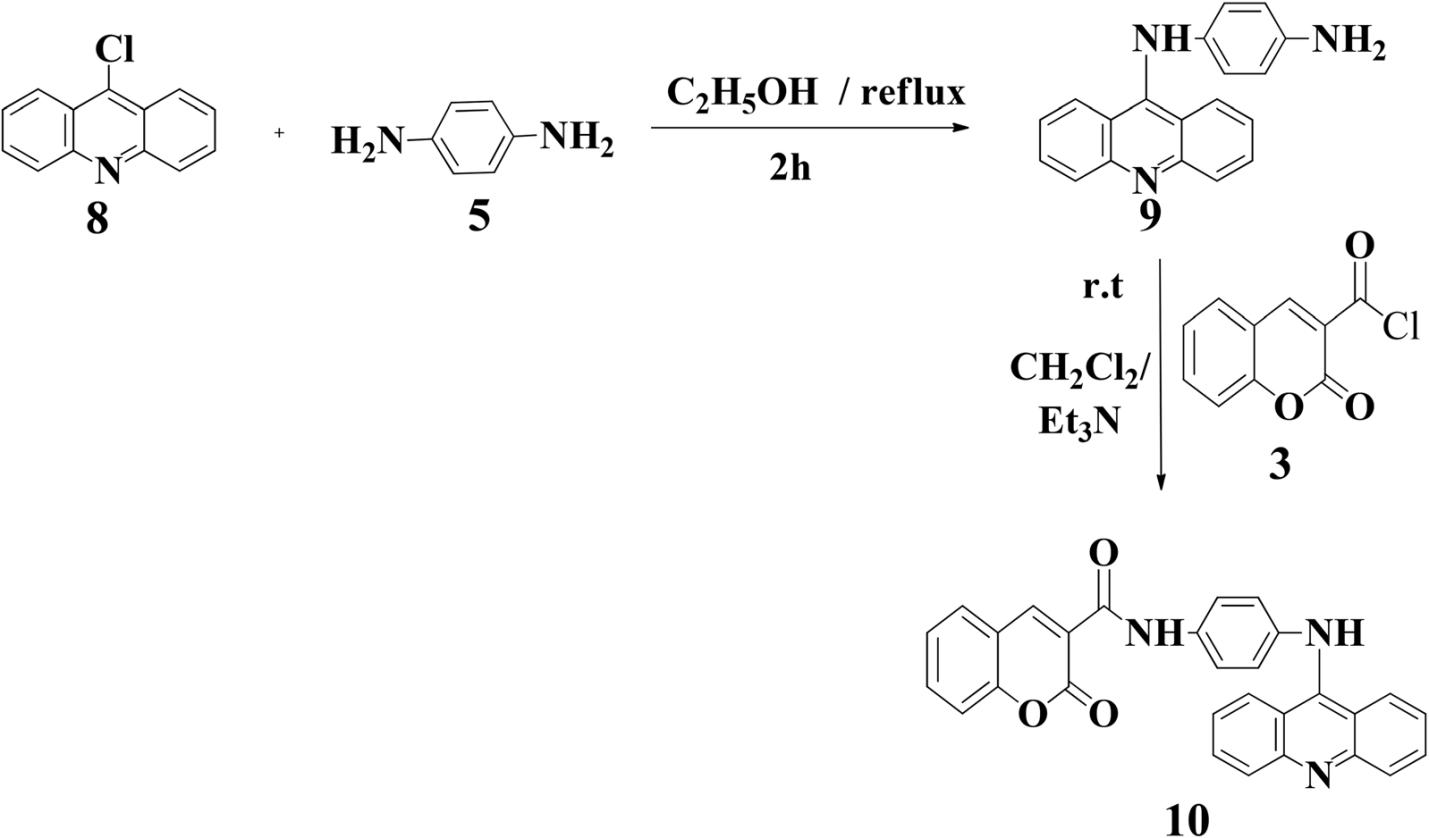
Production of coumarin-acridine hybrid 10 (drug B).

The preparation of the key intermediate 9-chloroacridine 8 (Scheme 3) needed for assembling drug A was done in two steps by a modified Ullman–Goldberg reaction of 2-chlorobenzoic acid with aniline in the presence of anhydrous potassium carbonate. Cu (acts as a catalyst) and CuO (acts as a co-catalyst), in DMF as the solvent, were subjected to heating at 125 °C to give intermediate *N*-phenylanthranilic acid, which was further cyclized under reflux conditions in phosphorus oxychloride (POCl_3_) to afford 9-chloroacridine 8, as depicted in Scheme S5 ref. 20 (SI). Moreover, the targeted hybrid 10 was assembled by the condensation of 9-chloroacridine 8 with bis-amine 5, affording the condensed monoamine 9, according to the reported method;^36,37^ further reaction with 3 in equimolar amounts in the presence of excess triethyl amine gave hybrid 10 in good yields (79.8%) as shown in Scheme 3.

**Scheme 4:**
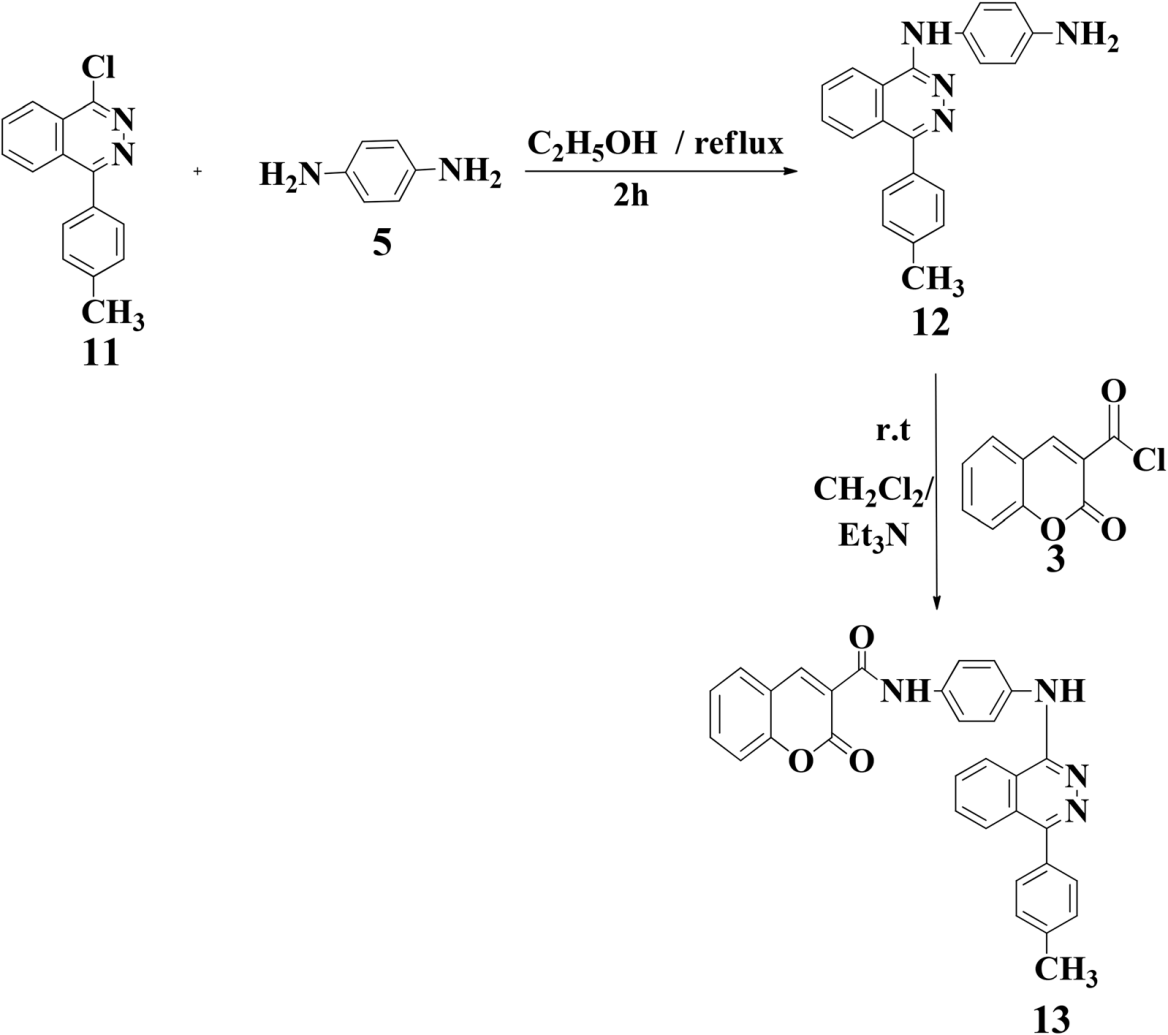
Synthesis of coumarin-phthalazine hybrid 13.

The preparation of 1-chloro-4-(*p*-tolyl) phthalazine 11 in Scheme 4 was achieved by following a previously published method,^21,22^ involving a Friedel–Crafts acylation reaction between toluene and phthalic anhydride using a catalytic amount of anhydrous aluminum chloride. Subsequent hydrazinolysis of the resulting acid with hydrazine hydrate in ethanol yielded 4-(4-tolyl)phthalazin-1(2*H*)-one, which was then treated with phosphorus oxychloride, resulting in the formation of 1-chloro-4-(4-tolyl)phthalazine 11 as shown in Schemes S5 and S6 (SI). This chlorinated phthalazine, key scaffold 11, served as a template intermediate for synthesizing a substituted phthalazine hybrid through reaction with a nitrogen-containing nucleophile. The synthetic schemes and the characterization data are presented in the supplementary material. Further condensation of 11 with 5 gave 12 in good yield, according to a method published elsewhere.^38,39^ Finally, the reaction of 12 with acid chloride 3 in equimolar amounts in the presence of extra triethylamine produced the corresponding coumarin-phthalazine hybrid 13 in good yield (75%) as revealed in Scheme 4. The advancement of the reaction was observed by TLC by applying hexane and ethanol (3 : 1) as an eluent mixture until the starting materials were fully consumed.

### 3.2. Fabrication of drug-loaded and unloaded PMMA/PVP nanofibrous scaffolds

All electrospinning solutions resulted in the successful production of nanofibrous scaffolds with well-formed fibers. The fabrication process proceeded smoothly and continuously, with stable Taylor cones observed throughout the electrospinning methods. Increasing drug concentration in the solutions enhanced the stretching of nanofibers, due to the improved homogeneity and conductivity of the polymers. Based on this observation, a final blended concentration of 5% for both drugs was selected, resulting in the production of uniform and continuous fibers with excellent quality.

#### 3.2.1. SEM investigation of PMMA/PVP NFs

**Fig. 1:**
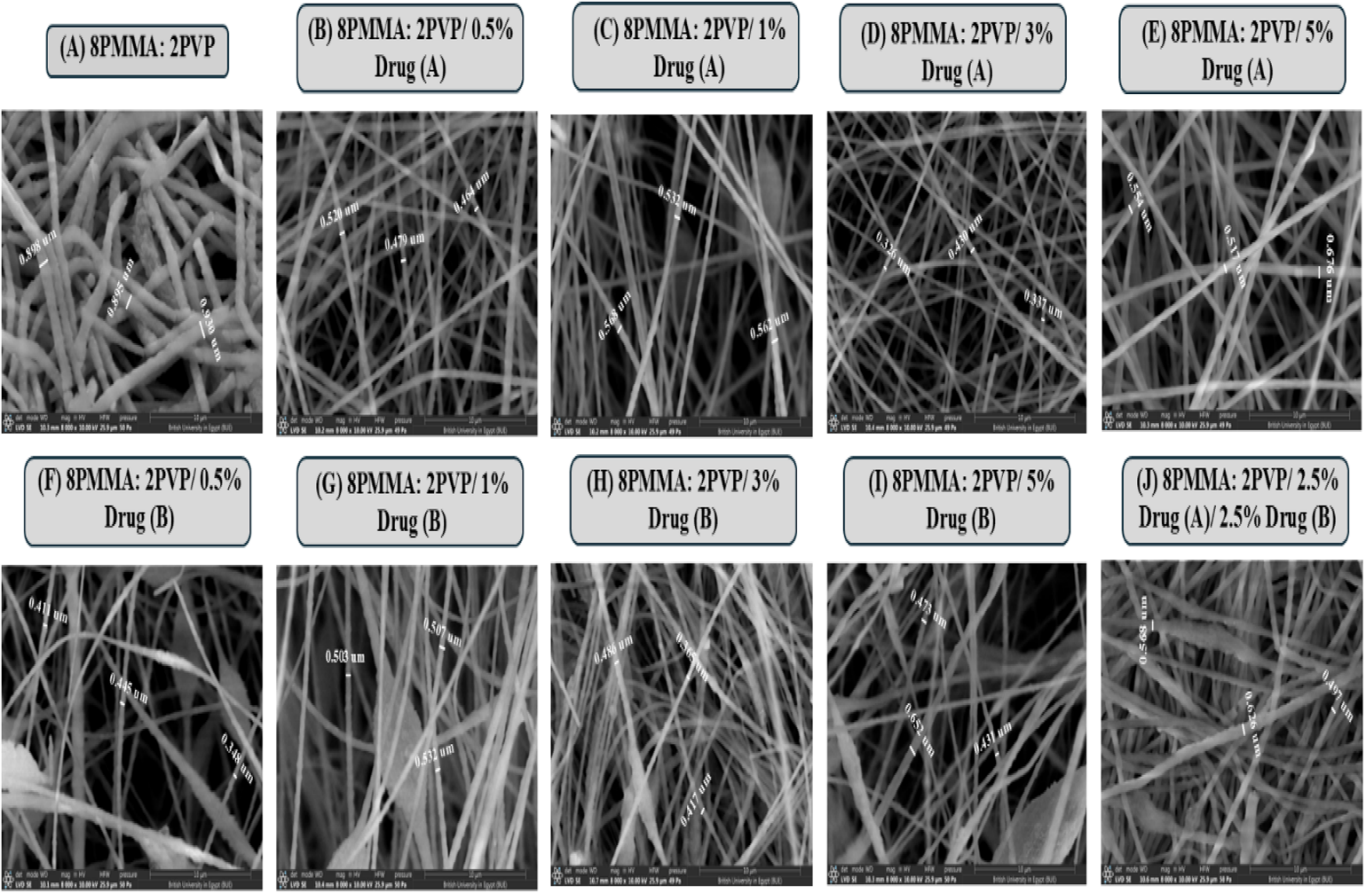
SEM micrographs of PMMA/PVP NFs. (A) (8PMMA: 2PVP), (B) (8PMMA: 2PVP/0.5% drug (A)), (C) (8PMMA: 2PVP/1% drug (A)), (D) (8PMMA: 2PVP/3% drug (A)), (E) (8PMMA: 2PVP/5% drug (A)), (F) (8PMMA: 2PVP/0.5% drug (B)), (G) (8PMMA: 2PVP/1% drug (B)), (H) (8PMMA: 2PVP/3% drug (B)), (I) (8PMMA: 2PVP/5% drug (B)), and (J) (8PMMA: 2PVP/2.5% drug (A)/2.5% drug (B)) (the mean diameter of unloaded NFs is 0.6–0.8 μm, and that of drug-loaded NFs is 0.4–0.6 μm, using Image-J software).

SEM was widely utilized to inspect the surface structure and morphology of nanofiber scaffolds, with optimization steps undertaken to determine the ideal drug concentrations for incorporating both drugs into the PMMA/PVP scaffold. As shown in Fig. 1, adding either drug (A) or (B) leads to a decrease in fabricated nanofiber diameters in comparison to the control (*i.e.*, drug-free NFs) Fig. 1A. This is attributed to the increased homogeneity, stretching, and enhanced conductivity of the solution. Moreover, it was observed that drug (B) formed more encapsulated beads than drug (A), which may be caused by the increased homogeneity and conductivity of drug (A) than drug (B).^40^ Notably, the addition of drugs enhanced the fiber matrix and increased the concentration of drugs in the solution, forming good-quality nanofibers. The final scaffold with the blended drugs and high drug concentration demonstrated good nanofibers with increased diameters, compared to drug-free NFs. Also, the two blended drugs led to increased homogeneity of the solution and decreased drug (B) bead formation, which was the result of drug encapsulation.^41^

#### 3.2.2. FTIR analysis of PMMA/PVP NFs

The PVP spectrum shows that the amide group's hybridization causes the observed peak shifts. These shifts occur due to reactions at the nitrogen atom of C=O. This is why the sharp peak at *ν* 1645 cm^−1^ corresponds to C=O stretching that is referred to as amide-I rather than solely a carbonyl stretch, particularly for amides. Several peaks are straightforward to assign, including the broad stretching at approximately *ν* 3400 cm^−1^ (O–H, stretch), *ν* 2900 cm^−1^ (C–H_*n*_, stretch), *ν* 1645 cm^−1^ (amide-I), and *ν* 1495–1425 cm^−1^ (C–H_*n*_, deformation). Another peak, at *ν* 1285 cm^−1^, has been associated with N–C stretching. The bands at *ν* 1425 and *ν* 1285 cm^−1^ are related to the flexible CH_2_ vibration and the C–N expanding vibration band, respectively. The hydrogen-bonded O–H extending mode of pure PVP was observed in the *ν* 3500–3200 cm^−1^ region.^42,43^

The PMMA spectrum exhibits distinct peaks, including a strong, sharp peak at *ν* 1730 cm^−1^, associated with CO stretching at *ν* 1147 cm^−1^, which is ascribed to the C–O bond within the ester group, and elongating vibrations of CH_3_ groups occurring at *ν* 2995 and 2951 cm^−1^. Other observed peaks at *ν* 1270 and 1240 cm^−1^ signify C–C–O bonds within the ester group, while those at *ν* 1475 and ∼1440 cm^−1^ signify skeletal CH_2_ deformations. Furthermore, peaks at *ν* 1186 and 1147 cm^−1^ are linked to C–O–C vibrations of the methoxy group. The FTIR spectrum of PMMA also shows bands at around *ν* 750 cm^−1^ arising from OH bending.^30,43^

**Fig. 2:**
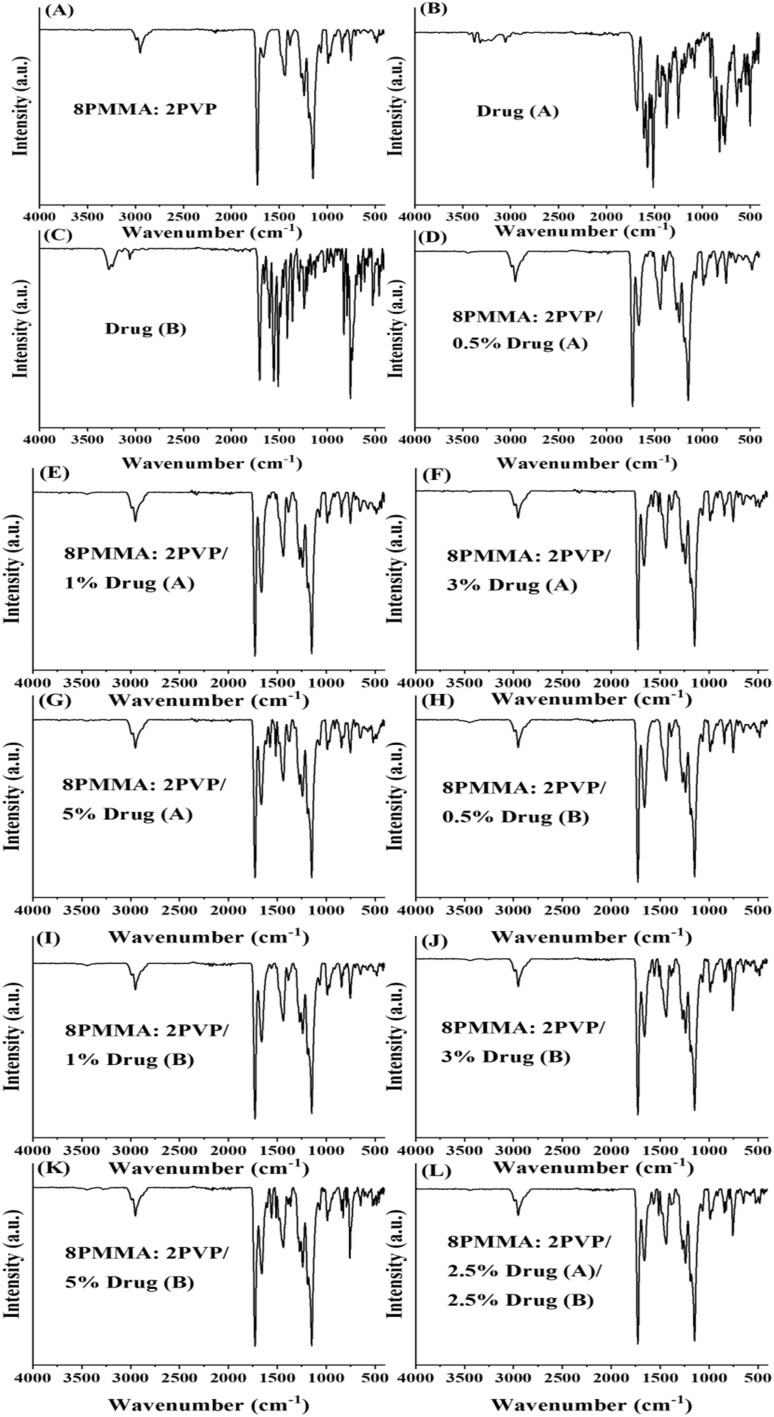
FTIR spectra of separate components of electrospun PMMA/PVP nanofiber scaffolds. (A) PVP, (B) (8PMMA: 2PVP), (C) drug (A), (D) drug (B), (E) (8PMMA: 2PVP/0.5% drug (A)), (F) (8PMMA: 2PVP/1% drug (A), (G) (8PMMA: 2PVP/3% drug (A)), (H) (8PMMA: 2PVP/5% drug (A)), (I) (8PMMA: 2PVP/0.5% drug (B)), (J) (8PMMA: 2PVP/1% drug (B)), (K) (8PMMA: 2PVP/3% drug (B)), (L) (8PMMA: 2PVP/5% drug (B)), and (M) (8PMMA: 2PVP/2.5% drug (A)/2.5% drug (B)).

FTIR spectra shown in Fig. 2 demonstrate the PVP/PMMA scaffold blend. These spectra exhibit strong peaks corresponding to PMMA and PVP, providing evidence for the creation of the blend. Thus, FTIR analysis serves to validate the formation of the polymer blend. Moreover, it was demonstrated in the IR results that the drugs were encapsulated inside the matrix, as the peaks of the drug did not appear in the blended mixture.

Coumarin-quinoline hybrid (drug A): the IR spectrum of coumarin-quinoline typically exhibits characteristic absorption peaks corresponding to various functional groups present within the molecule. The amide C=O stretch, indicative of the carbonyl functionality in amide groups, is commonly observed at *ν* 1650–1700 cm^−1^. Aromatic C=C stretches, associated with double bonds in aromatic rings, typically appear at *ν* 1600–1500 cm^−1^. Quinoline ring vibrations, specific to compounds containing quinoline moieties, might manifest as distinct bands at *ν* 1700–1500 cm^−1^. Aromatic C–H stretching, representative of hydrogen atoms bonded to aromatic carbon atoms, is usually observed in the vicinity of *ν* 3100–3000 cm^−1^. C–O stretching in carbonyl groups, characteristic of ester or amide functionalities, is often present at *ν* 1200–1000 cm^−1^. Additionally, C–N stretching, indicative of carbon-nitrogen bonds, is expected to occur at *ν* 1300–1000 cm^−1^.

Coumarin-acridine hybrid (drug B): the amide C=O stretch, denoting the presence of carbonyl groups in amides, typically occurs between *ν* 1650–1700 cm^−1^. The amide N–H stretch, indicative of hydrogen bonding in amides, is commonly observed at *ν* 3300–3500 cm^−1^. Aromatic C=C stretches, corresponding to double bonds within aromatic rings, are expected in the range of *ν* 1600–1500 cm^−1^. Vibrations specific to the acridine ring, characteristic of compounds containing acridine moieties, may manifest as distinct bands around *ν* 1650–1500 cm^−1^. Aromatic C–H stretching, associated with hydrogen atoms bonded to aromatic carbon atoms, is typically observed within the region of *ν* 3100–3000 cm^−1^. C–O stretching in carbonyl groups, found in esters and amides, is present around *ν* 1200–1000 cm^−1^. Additionally, the C–N stretch representing carbon-nitrogen bonds is expected to occur within the range of *ν* 1300–1000 cm^−1^. These characteristic IR peaks provide valuable information for the structural elucidation and analysis of organic compounds.^3^

### 3.3. Bio-evaluation tests

#### 3.3.1. *In vitro* anticancer assessment

##### 3.3.1.1. Antiproliferative activity

**Fig. 3:**
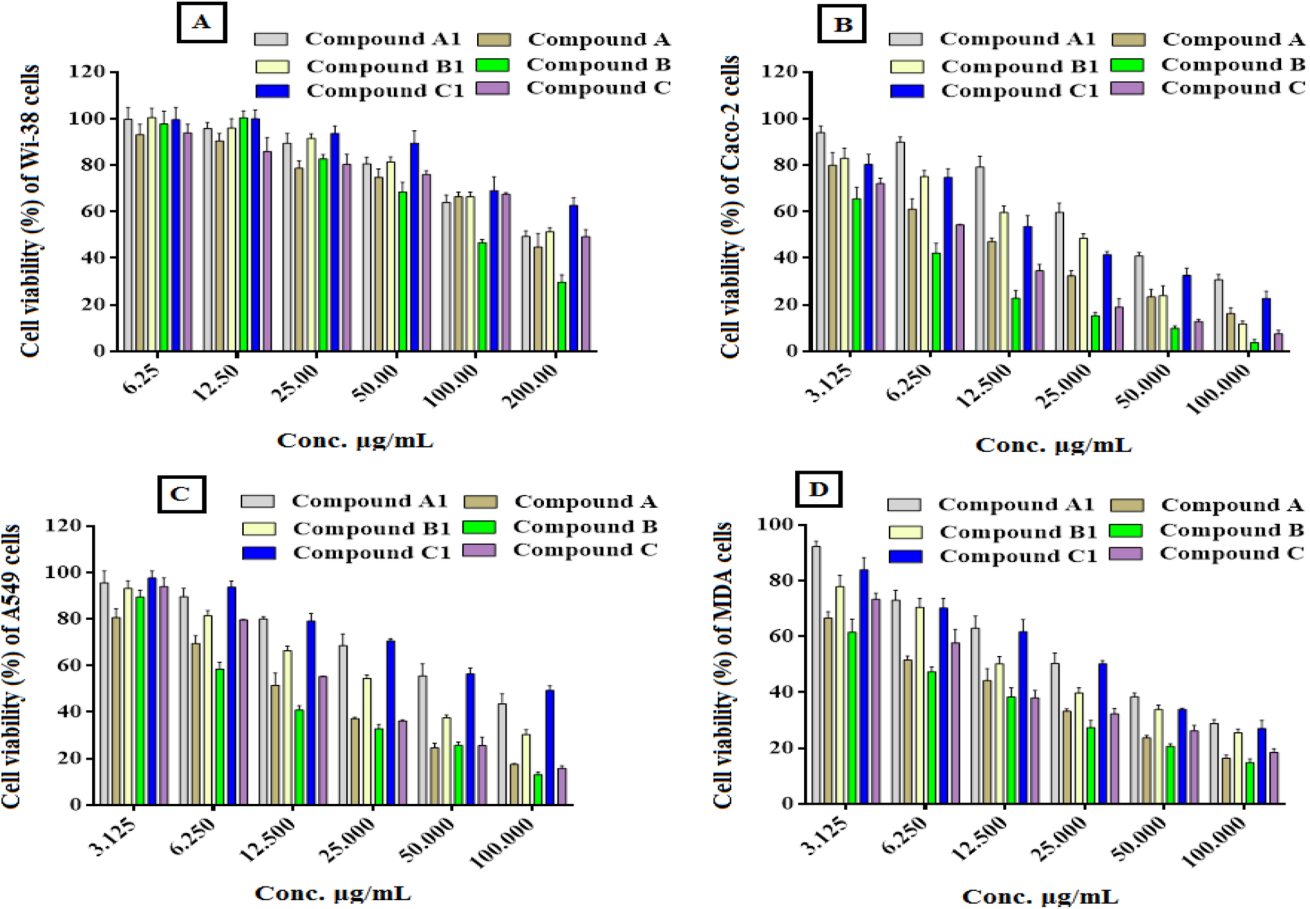
*In vitro* anti-proliferation effects of the synthesized compounds on the normal and cancer cell lines after 48 h of incubation. Wi-38 cells (A), Caco-2 cells (B), A431 cells (C) and MDA cells (D) were incubated with the synthesized compounds at different concentrations, and the cytotoxic effect was assessed using the MTT assay. All values are stated as (mean ± SD) and denote the average values from three tests.

The anticancer activities of the newly manufactured derivatives (compounds coded A1, A, B1, B, C1, and C) were assessed *in vitro* alongside diverse kinds of human cancer cells, including Caco-2, A549, and MDA cell lines, compared to the healthy human lung fibroblast (Wi-38) cells. Herein, the IC_50_ of the manufactured derivatives against Wi-38 cells was found to extend from 126.3 to 195.0 μg mL^−1^ (Table 1), revealing a prominent level of safety for these synthesized compounds on the treated human normal (Wi-38) cell line. Conversely, these synthesized derivatives demonstrated a high selectivity toward treated Caco-2, A549, and MDA cells with low IC_50_. Our results reveal that all treated cancer cell lines were more sensitive to derived compounds (A, B, and C) than the precursor compounds (A1, B1, and C1). Furthermore, the precursor compound of B and its derivative B1 showed potent anticancer activity against all tested cells, more than other tested compounds (A1, A, C1, and C). The anticancer activities of the synthesized compounds showed a dose dependence on all the treated tumor cell lines, as shown in Fig. 3. The IC_50_ values of these potent derivatives against Caco-2 cells were estimated to range from 4.87 to 38.23 μg mL^−1^, with SI values ranging from 4.59 to 25.93. Their IC_50_ values against A549 cells were estimated to range from 5.74 to 32.05 μg mL^−1^ with SI values ranging from 5.47 to 18.09. However, their IC_50_ values against MDA cells were estimated to extend from 4.27 to 14.91 μg mL^−1^, with SI values ranging from 11.76 to 29.58 (Table 1). The synthesized derivative B showed the most powerful anticancer activity with the greatest selectivity and lowest IC_50_ values.

**Table 1:** Cytotoxicity and anticancer effects of the synthesized compounds against Caco-2, A549 and MDA cells, compared with normal human (Wi-38) cells, stated in IC_50_ (μg mL^−1^) and SI values^a^

Compounds	Wi-38	Caco-2		A549		MDA	
	IC_50_	IC_50_	SI	IC_50_	SI	IC_50_	SI
Compound A1	175.3 ± 14.08	38.23 ± 1.64	4.59 ± 0.37	32.05 ± 2.18	5.47 ± 0.44	14.91 ± 0.96	11.76 ± 0.94
Compound A	138.9 ± 12.04	9.03 ± 1.53	15.38 ± 1.33	11.47 ± 1.75	12.11 ± 1.05	5.46 ± 0.27	25.44 ± 2.21
Compound B1	195.0 ± 14.41	24.15 ± 1.76	8.07 ± 0.59	16.93 ± 1.88	11.52 ± 0.85	8.92 ± 0.67	21.86 ± 1.62
Compound B	126.3 ± 10.95	4.87 ± 1.69	25.93 ± 2.25	6.98 ± 1.99	18.09 ± 1.57	4.27 ± 0.46	29.58 ± 2.56
Compound C1	192.7 ± 16.79	12.09 ± 1.42	15.94 ± 1.39	21.01 ± 2.05	9.17 ± 0.79	13.98 ± 0.57	13.78 ± 1.21
Compound C	136.9 ± 11.67	7.11 ± 1.68	19.25 ± 1.36	12.17 ± 1.36	11.25 ± 1.04	6.06 ± 0.81	22.59 ± 1.93
5-FU	5.23 ± 0.18	6.13 ± 0.28	0.85 ± 0.03	5.74 ± 0.24	0.92 ± 0.03	5.89 ± 0.19	0.89 ± 0.02

a Data are presented as mean ± SD.

##### 3.3.1.2. *In vitro* anti-migration activity

**Fig. 4:**
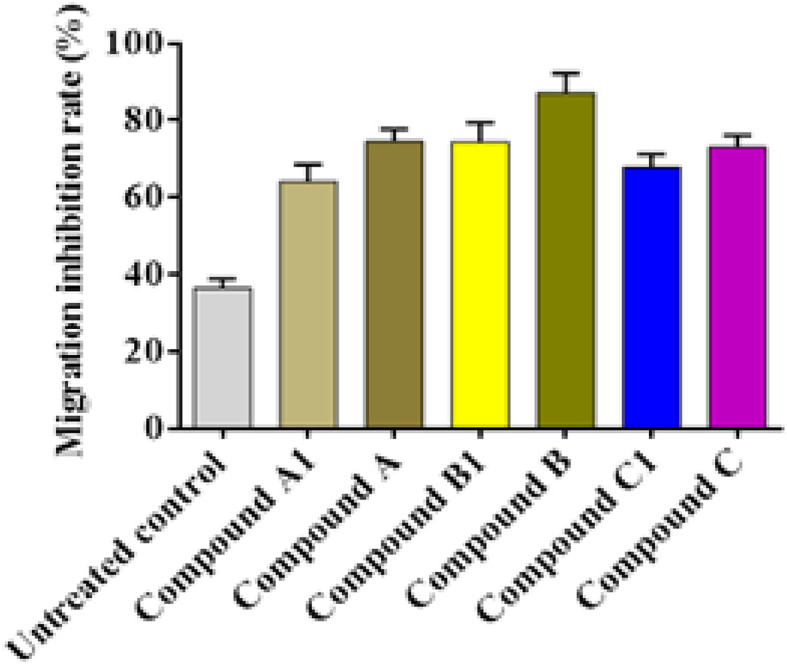
Anti-migration efficacy of the newly synthesized derivatives *via* wound healing scratch assay showing relative migration inhibition percentages. Values are presented as mean ± S.D.

Most significantly, the anti-metastatic action of all tested derivatives demonstrated inhibition potency against the cell migration of the treated A549 cells. The synthesized compound B exhibited the most potent antimigration activity of 87.21%, followed by the synthesized compounds coded B1 and C, which inhibited the cell migration by 74.63% and 74.45%, respectively. The synthetic compounds coded A1, C, and compound C1, correspondingly reduced the migration of A549 cells by 64.25%, 79.09%, and 67.89% respectively, as shown in Fig. 4.

#### 3.3.2. Antimicrobial activity assessment

##### 3.3.2.1. Antimicrobial potency of drug-unloaded-PMMA/PVA nanofibrous scaffolds

**Fig. 5:**
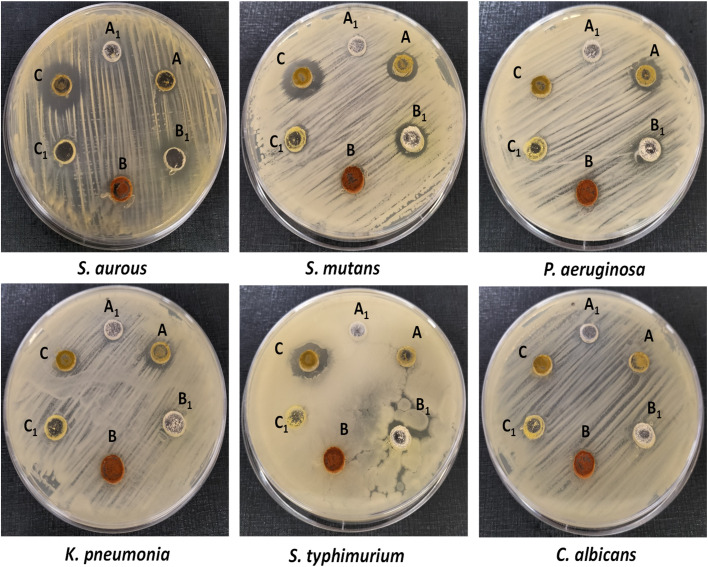
Antimicrobial activity of the synthesized compounds against several human pathogens, using agar well diffusion methods; *A*_1_, B_1_, and C_1_ represent the precursor compounds for the three prepared compounds A, B, and C, respectively.

Recently, the extent of multidrug-resistant microbial pathogens has presented a serious health challenge that has largely affected the entire world.^44,45^ Several approaches have been proposed to alleviate the crisis by controlling antibiotic misuse and developing new antimicrobial agents with lower resistance-induction potential.^46,47^ Thus, the antimicrobial effects of the synthesized candidates were assessed toward several human pathogens through the agar-well diffusion technique. The results in Fig. 5 show the wide-spectrum antibacterial activity of compounds A and C against the applied bacterial pathogens. Compound C reveals potent antimicrobial activity toward Gram-positive bacteria, compared to Gram-negative ones, whereas the highest antimicrobial activity was about 19.5 ± 2.3 and 17.2 ± 1.3 mm toward *S. aureus* and *S. mutans,* respectively, followed by *S. typhimurium* (about 15.5 ± 1.1 mm). The outer envelope structure in Gram-negative bacteria is exploited as a physical barrier and hence greatly enhances their drug resistance compared to Gram-positive pathogens.^48,49^ Compound A showed equal antimicrobial activity (about 14 mm) against *S. mutans*, *K. pneumonia*, and *P. aeruginosa.* Interestingly, compound B revealed no antimicrobial activity against all applied pathogens, whereas its precursor (B_1_) revealed considerable antimicrobial activity alongside the two applied Gram-positive bacteria, *S. aureus* and *S. mutans*, of about 13 mm. Additionally, the prepared compounds revealed no antifungal activity against *C. albicans.*

##### 3.3.2.2. Antimicrobial potency of drug-loaded-PMMA/PVA nanofibrous scaffolds

**Fig. 6:**
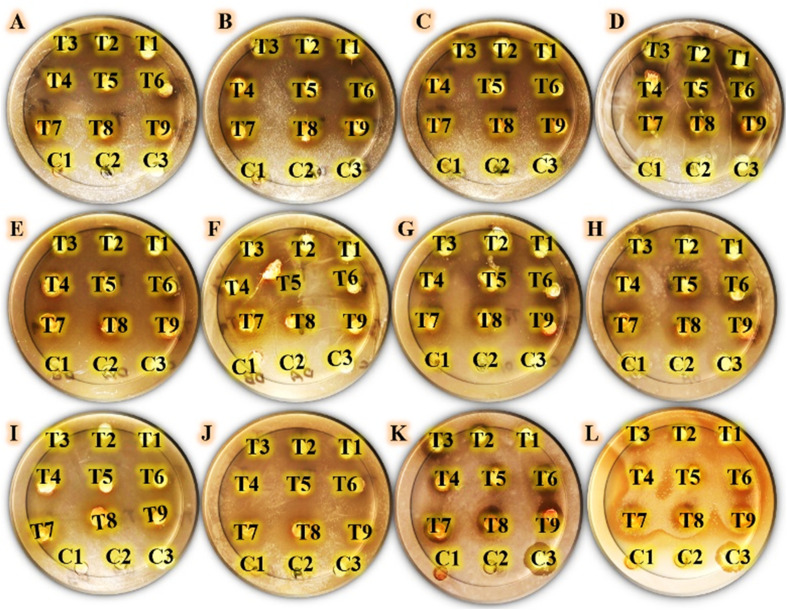
Agar-well diffusion results indicate the antimicrobial capabilities of the tested formulations. T1: (8 PMMA, 2 PVP, and 0.5% drug A), T2: (8 PMMA, 2 PVP, and 1% drug A), T3: (8 PMMA, 2 PVP, and 3% drug A), T4: (8 PMMA, 2 PVP, and 5% drug A), T5: (8 PMMA, 2 PVP, and 0.5% drug B), T6: (8 PMMA, 2 PVP, and 1% drug B), T7: (8 PMMA, 2 PVP, and 3% drug B), T8: (8 PMMA, 2 PVP, and 5% drug B), and T9: (8 PMMA, 2 PVP, 2.5% drug A, and 2.5% drug B), compared with tested controls C1: (8 PMMA and 2 PVP), C2: (0.1% drug A), and C3: (0.1% drug B). This assay was recorded against the tested human pathogens, coded as follows. (A): *Salmonella paratyphi*, (B): *Escherichia coli*, (C): *Klebsiella pneumoniae*, (D): *Pseudomonas aeruginosa*, (E): *Staphylococcus epidermidis*, (F): *Staphylococcus aureus*, (G): *Bacillus cereus*, (H): *Bacillus subtilis*, (I): *Candida krusei*, (J): *Candida glabrata*, (K): *Candida albicans*, and (L): *Candida parapsilosis*.

**Fig. 7:**
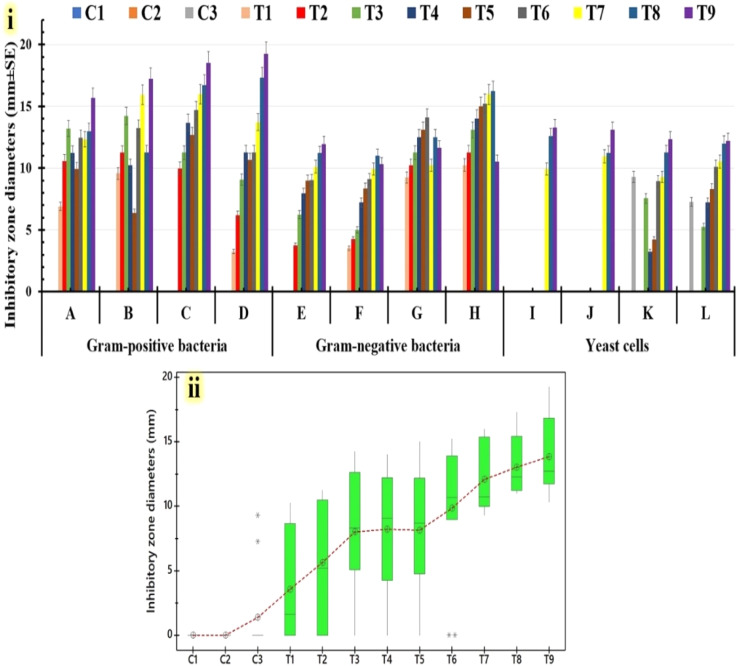
Inhibitory widths produced by the tested formulations. T1: (8 PMMA, 2 PVP, and 0.5% drug A), T2: (8 PMMA, 2 PVP, and 1% drug A), T3: (8 PMMA, 2 PVP, and 3% drug A), T4: (8 PMMA, 2 PVP, and 5% drug A), T5: (8 PMMA, 2 PVP, and 0.5% drug B), T6: (8 PMMA, 2 PVP, and 1% drug B), T7: (8 PMMA, 2 PVP, and 3% drug B), T8: (8 PMMA, 2 PVP, and 5% drug B), and T9: (8 PMMA, 2 PVP, 2.5% drug A, and 2.5% drug B), compared with tested controls C1: (8 PMMA and 2 PVP), C2: (0.1% drug A), and C3: (0.1% drug B). These antimicrobial activities were measured against several multidrug-resistant human pathogens *via* the agar-well diffusion test, labeled as follows. (A): *Salmonella paratyphi*, (B): *Escherichia coli*, (C): *Klebsiella pneumoniae*, (D): *Pseudomonas aeruginosa*, (E): *Staphylococcus epidermidis*, (F): *Staphylococcus aureus*, (G): *Bacillus cereus*, (H): *Bacillus subtilis*, (I): *Candida krusei*, (J): *Candida glabrata*, (K): *Candida albicans*, and (L): *Candida parapsilosis*. Chart showing calculated inhibition zones (i). The Tukey–Kramer post-hoc analysis box-plot graph shows the inhibitory value distributions corresponding to the tested formulations (ii).

Initially, the antimicrobial capacity of the estimated formulations was assessed by the agar-well-diffusion method. Fig. 6 shows the antimicrobial evaluation of the tested formulations by determining the inhibitory zone widths that were generated against human pathogens. Compared to Gram-negative bacteria and yeast cells, Gram-positive bacteria were the target of the largest inhibitory zone widths (Fig. 7i). The Tukey–Kramer post-hoc analysis is used to prove that the inhibitory value distributions are parallel for the examined formulations. Box-plot graphs of all human pathogens under study (Fig. 7ii) indicate that there are minor distinctions between T1 and T2 formulations. It was also observed that there were small variations in the T3, T4, and T5 formulations. Furthermore, there exist notable differences in T6, T7, and T8 compositions. The results indicate that the T9 formula produces the highest inhibitory zone widths (Fig. 7ii). Table 2 shows that neither C1 nor C2 controls have any effect on any of the human infections investigated. Additionally, the C3-control only has an impact on a few examined yeast infections, namely *Candida albicans* and *Candida parapsilosis*. Remarkably, the T6, T7, and T8 compositions have the highest inhibitory values (15.22 ± 3.69, 15.96 ± 1.58, and 16.23 ± 1.11 mm, respectively) alone against *Pseudomonas aeruginosa* among the Gram-negative bacteria that were examined. Compared to the other human pathogens under investigation, the T9 formula produces the largest inhibitory zones. Also, the T9 formula shows the highest inhibitory zone width against *Bacillus subtilis* (19.25 ± 2.12 mm), followed by *Candida krusei* (13.26 ± 1.15 mm) and *Salmonella paratyphi* (11.96 ± 3.47 mm).

**Table 2:** Antimicrobial efficacy results for the tested formulations, labeled as follows. T1: (8 PMMA, 2 PVP, and 0.5% drug A), T2: (8 PMMA, 2 PVP, and 1% drug A), T3: (8 PMMA, 2 PVP, and 3% drug A), T4: (8 PMMA, 2 PVP, and 5% drug A), T5: (8 PMMA, 2 PVP, and 0.5% drug B), T6: (8 PMMA, 2 PVP, and 1% drug B), T7: (8 PMMA, 2 PVP, and 3% drug B), T8: (8 PMMA, 2 PVP, and 5% drug B), and T9: (8 PMMA, 2 PVP, 2.5% drug A, and 2.5% drug B), compared with tested controls C1: (8 PMMA and 2 PVP), C2: (0.1% drug A), and C3: (0.1% drug B), measured *versus* many multidrug-resistant human pathogens by the agar-well diffusion test

Multidrug-resistant human pathogens	Inhibition zone diameters (mm ± SD)^a^											
	C1	C2	C3	T1	T2	T3	T4	T5	T6	T7	T8	T9
*Staphylococcus epidermidis*	0	0	0	6.89 ± 0.35^ef^	10.59 ± 2.44^de^	13.21 ± 3.78^cd^	11.23 ± 3.87 ^cd^	9.95 ± 1.11 ^cd^	12.45 ± 2.58^bc^	12.33 ± 0.68^ab^	12.98 ± 3.54^a^	15.67 ± 3.94^a^
*Staphylococcus aureus*	0	0	0	9.57 ± 1.14^ef^	11.25 ± 1.12^de^	14.23 ± 2.98^cd^	10.21 ± 3.66^cd^	6.37 ± 1.03 ^cd^	13.25 ± 2.54^bc^	15.92 ± 3.54^ab^	11.27 ± 3.22^a^	17.23 ± 3.41^a^
*Bacillus cereus*	0	0	0	0	9.99 ± 0.97^de^	11.25 ± 0.98^cd^	13.67 ± 3.47^cd^	12.67 ± 2.54^cd^	14.67 ± 3.54^bc^	15.99 ± 2.22^ab^	16.72 ± 3.47^a^	18.51 ± 0.54^a^
*Bacillus subtilis*	0	0	0	3.25 ± 1.20^ef^	6.19 ± 0.34^de^	9.07 ± 2.89 ^cd^	11.26 ± 1.12^cd^	10.66 ± 1.17^cd^	11.26 ± 0.97^bc^	13.71 ± 1.23^ab^	17.29 ± 2.11^a^	19.25 ± 2.12^a^
*Salmonella paratyphi*	0	0	0	0	3.75 ± 1.12^de^	6.25 ± 1.09^cd^	7.96 ± 0.22^cd^	8.99 ± 0.52^cd^	9.02 ± 1.15^bc^	10.11 ± 2.33^ab^	11.21 ± 1.99^a^	11.96 ± 3.47^a^
*Escherichia coli*	0	0	0	3.52 ± 0.39^ef^	4.25 ± 0.98d^e^	5.01 ± 0.33^cd^	7.22 ± 1.54^cd^	8.36 ± 1.15^cd^	9.11 ± 3.54^bc^	9.92 ± 3.54^ab^	10.99 ± 2.47^a^	10.32 ± 1.09^a^
*Klebsiella pneumoniae*	0	0	0	9.25 ± 1.34^ef^	10.21 ± 0.78^de^	11.25 ± 1.25^cd^	12.51 ± 3.54 ^cd^	13.09 ± 4.06^cd^	14.11 ± 2.59^bc^	10.23 ± 0.99^ab^	12.52 ± 3.58^a^	11.66 ± 1.87^a^
*Pseudomonas aeruginosa*	0	0	0	10.25 ± 1.14^ef^	11.26 ± 1.89^de^	13.09 ± 1.98^cd^	14.02 ± 0.98^cd^	14.99 ± 0.22^cd^	15.22 ± 3.69^bc^	15.96 ± 1.58^ab^	16.23 ± 1.11^a^	10.55 ± 1.97^a^
*Candida krusei*	0	0	0	0	0	0	0	0	0	9.92 ± 3.19^ab^	12.59 ± 2.59^a^	13.26 ± 1.15^a^
*Candida glabrata*	0	0	0	0	0	0	0	0	0	10.95 ± 1.58^ab^	11.22 ± 2.58^a^	13.09 ± 3.54^a^
*Candida albicans*	0	0	9.29 ± 1.25^fg^	0	0	7.56 ± 0.88 ^cd^	3.25 ± 0.54 ^cd^	4.23 ± 1.58 ^cd^	8.95 ± 2.54^bc^	9.29 ± 2.14^ab^	11.27 ± 1.89^a^	12.33 ± 2.26^a^
*Candida parapsilosis*	0	0	7.25 ± 2.33^fg^	0	0	5.27 ± 0.14 ^cd^	7.21 ± 0.74 ^cd^	8.32 ± 1.54 ^cd^	10.12 ± 1.54^bc^	10.52 ± 0.14^ab^	11.99 ± 2.94^a^	12.21 ± 1.23^a^

a The data are shown as the mean (millimeters) ± standard deviation (mm ±SD). The differences are statistically significant at *p* ≤ 0.05. Therefore, statistically significant differences (*p* ≤ 0.05) are indicated by differences in the superscript letters (a, b, c, d, e, f, and g).

**Fig. 8:**
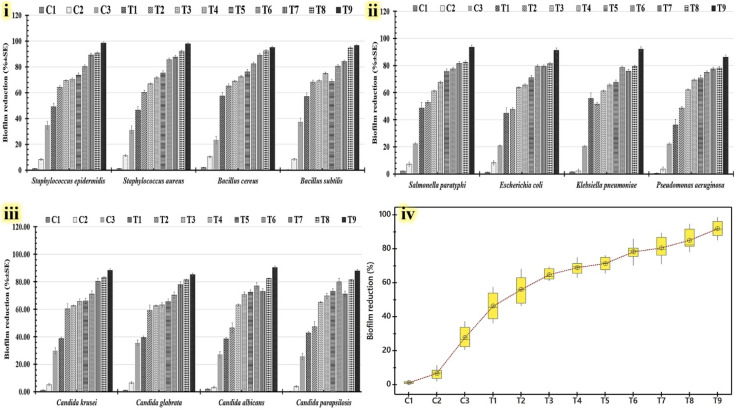
Reduction in biofilm generation of tested human pathogens, including *Salmonella paratyphi*, *Escherichia coli*, *Klebsiella pneumoniae*, *Pseudomonas aeruginosa*, *Staphylococcus epidermidis*, *Staphylococcus aureus*, *Bacillus cereus*, *Bacillus subtilis*, *Candida krusei*, *Candida glabrata*, *Candida albicans*, and *Candida parapsilosis* that were treated with all tested formulations labeled T1: (8 PMMA, 2 PVP, and 0.5% drug A), T2: (8 PMMA, 2 PVP, and 1% drug A), T3: (8 PMMA, 2 PVP, and 3% drug A), T4: (8 PMMA, 2 PVP, and 5% drug A), T5: (8 PMMA, 2 PVP, and 0.5% drug B), T6: (8 PMMA, 2 PVP, and 1% drug B), T7: (8 PMMA, 2 PVP, and 3% drug B), T8: (8 PMMA, 2 PVP, and 5% drug B), and T9: (8 PMMA, 2 PVP, 2.5% drug A, and 2.5% drug B), compared with tested controls C1: (8 PMMA and 2 PVP), C2: (0.1% drug A), and C3: (0.1% drug B). The chart shows the percentage of biofilm reduction for Gram-positive bacteria (i), Gram-negative bacteria (ii), and yeast cells (iii). A Tukey-post hoc analysis was conducted to compare biofilm reduction across all drug dosage groups (iv).

**Fig. 9:**
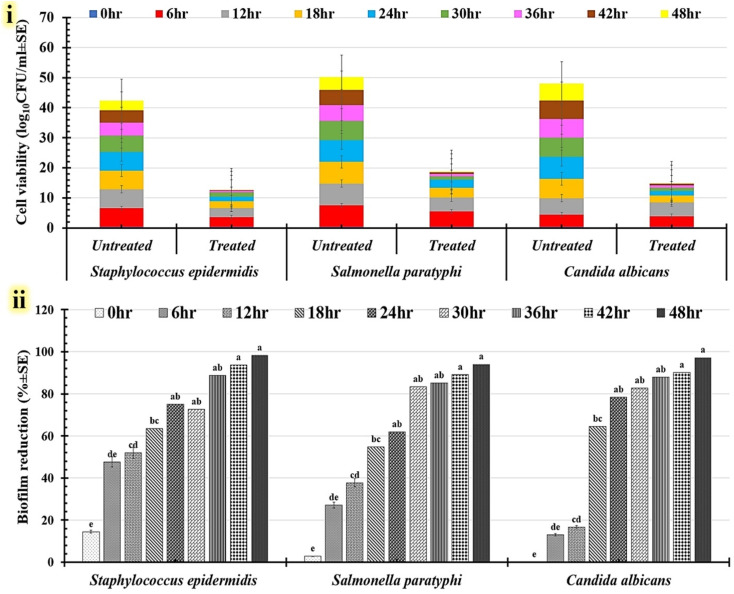
Multidrug-resistant human pathogens treated with the T9 formula and untreated (T9-formula-free) cells are displayed against reduction in cell viability (i), and the percentage biofilm reduction (ii) throughout the course of the incubation period.

A biofilm inhibition test resulting from the utilization of the micro-dilution technique was also performed to investigate the possibility that the assessed formulations could prevent the growth of active microbes. The Tukey–Kramer post-hoc analysis mean value for all tested formulations compared to the tested controls indicates that the T9 formula had the highest value of all tested formulations (Table 3). Additional data on the statistical clustering of anti-biofilm (%) for different examined formulations is displayed in a box plot graph (Fig. 8iv). The T9 formula deviates considerably from the control group and other analyzed formulations. In conclusion, the findings demonstrate that the anti-biofilm capabilities of the T9 formula are statistically significant. All examined human pathogens' capacity to produce biofilms was shown to be most significantly reduced when using the T9 formula (Fig. 8). This formula reduces the biofilms of Gram-positive bacteria (Fig. 9i), Gram-negative bacteria (Fig. 8ii), and yeast cells (Fig. 8iii). Table 2 shows that the highest anti-biofilm (%) was detected against *Staphylococcus epidermidis* (98.54 ± 3.56%), followed by *Salmonella paratyphi* (93.45 ± 2.54%) and *Candida albicans* (90.31 ± 2.98%).

**Table 3:** Antimicrobial efficacy results for tested formulations labeled as follows. T1: (8 PMMA, 2 PVP, and 0.5% drug A), T2: (8 PMMA, 2 PVP, and 1% drug A), T3: (8 PMMA, 2 PVP, and 3% drug A), T4: (8 PMMA, 2 PVP, and 5% drug A), T5: (8 PMMA, 2 PVP, and 0.5% drug B), T6: (8 PMMA, 2 PVP, and 1% drug B), T7: (8 PMMA, 2 PVP, and 3% drug B), T8: (8 PMMA, 2 PVP, and 5% drug B), and T9: (8 PMMA, 2 PVP, 2.5% drug A, and 2.5% drug B), compared with tested controls C1: (8 PMMA and 2 PVP), C2: (0.1% drug A), and C3: (0.1% drug B), measured *versus* against multidrug-resistant human pathogens by the biofilm inhibition test^a^

Multidrug-resistant human pathogens	Biofilm reduction (% ± SD)											
	C1	C2	C3	T1	T2	T3	T4	T5	T6	T7	T8	T9
*Staphylococcus epidermidis*	1.13 ± 0.33^j^	8.29 ± 0.19^i^	34.71 ± 1.56^h^	49.09 ± 5.47^g^	64.05 ± 4.22^f^	69.41 ± 3.03^e^	70.40 ± 3.22^d^	73.62 ± 2.85^d^	80.32 ± 6.54^c^	89.43 ± 1.98^c^	90.75 ± 2.65^b^	98.54 ± 3.56^a^
*Staphylococcus aureus*	1.09 ± 0.79^j^	11.31 ± 0.17^i^	31.25 ± 2.98^h^	46.53 ± 0.84^g^	60.24 ± 1.97^f^	67.01 ± 0.87^e^	71.55 ± 3.52^d^	75.36 ± 3.64^d^	85.74 ± 2.51^c^	87.68 ± 1.12^c^	92.05 ± 3.56^b^	97.98 ± 5.63^a^
*Bacillus cereus*	1.96 ± 0.04^j^	10.32 ± 0.19^i^	23.36 ± 2.96^h^	57.42 ± 4.89^g^	65.27 ± 2.14^f^	68.90 ± 4.16^e^	72.37 ± 0.63^d^	76.15 ± 5.59^d^	82.39 ± 2.54^c^	89.07 ± 2.64^c^	92.55 ± 1.92^b^	95.09 ± 2.06^a^
*Bacillus subtilis*	0.38 ± 0.17^j^	8.41 ± 0.31^i^	37.29 ± 2.84^h^	57.09 ± 3.20^g^	68.18 ± 1.74^f^	69.35 ± 2.19^e^	74.97 ± 0.61^d^	68.66 ± 2.55^d^	80.55 ± 1.14^c^	84.05 ± 2.86^c^	94.75 ± 3.67^b^	96.55 ± 2.54^a^
*Salmonella paratyphi*	2.03 ± 0.22^j^	7.24 ± 0.38^i^	22.55 ± 1.87^h^	48.66 ± 1.36^g^	52.81 ± 3.19^f^	61.17 ± 0.95^e^	67.56 ± 1.12^d^	75.84 ± 1.58^d^	77.59 ± 3.21^c^	81.58 ± 2.56^c^	82.36 ± 4.63^b^	93.45 ± 2.54^a^
*Escherichia coli*	1.17 ± 0.68^j^	8.31 ± 0.75^i^	20.93 ± 3.64^h^	44.78 ± 3.42^g^	47.79 ± 0.94^f^	63.84 ± 0.79^e^	65.61 ± 2.36^d^	70.97 ± 1.06^d^	79.41 ± 1.56^c^	79.29 ± 1.85^c^	81.45 ± 1.28^b^	91.30 ± 2.41^a^
*Klebsiella pneumoniae*	1.43 ± 0.26^j^	2.03 ± 0.77^i^	20.56 ± 2.65^h^	55.68 ± 3.54^g^	51.57 ± 3.45^f^	61.31 ± 2.97^e^	65.61 ± 4.97^d^	67.53 ± 1.52^d^	78.59 ± 3.54^c^	75.84 ± 1.15^c^	79.31 ± 0.98^b^	92.05 ± 0.79^a^
*Pseudomonas aeruginosa*	0.52 ± 0.26^j^	3.65 ± 0.47^i^	22.35 ± 2.58^h^	36.21 ± 3.99^g^	48.66 ± 1.11^f^	62.16 ± 0.77^e^	69.28 ± 1.78^d^	70.82 ± 3.83^d^	75.01 ± 0.98^c^	77.50 ± 0.61^c^	78.18 ± 3.21^b^	86.13 ± 0.25^a^
*Candida krusei*	1.11 ± 0.97^j^	5.24 ± 1.22^i^	29.91 ± 2.44^h^	38.65 ± 1.87^g^	60.25 ± 3.47^f^	62.68 ± 0.65^e^	65.61 ± 3.49^d^	66.15 ± 1.45^d^	71.12 ± 2.94^c^	80.32 ± 2.11^c^	83.29 ± 5.81^b^	88.28 ± 0.95^a^
*Candida glabrata*	1.04 ± 0.13^j^	6.48 ± 1.87^i^	35.48 ± 2.58^h^	39.45 ± 3.87^g^	59.21 ± 4.98^f^	62.59 ± 3.16^e^	63.19 ± 3.46^d^	65.61 ± 2.54^d^	70.29 ± 3.41^c^	77.86 ± 2.40^c^	81.58 ± 3.24^b^	85.27 ± 3.54^a^
*Candida albicans*	1.91 ± 0.24^j^	3.21 ± 0.99^i^	27.13 ± 3.96^h^	38.52 ± 6.78^g^	46.53 ± 0.98^f^	63.24 ± 2.56^e^	70.85 ± 2.32^d^	72.35 ± 1.98^d^	76.98 ± 3.19^c^	73.02 ± 3.52^c^	82.36 ± 3.54^b^	90.31 ± 2.98^a^
*Candida parapsilosis*	0.17 ± 0.60^j^	3.87 ± 0.14^i^	25.79 ± 1.98^h^	42.81 ± 5.38^g^	47.32 ± 0.77^f^	65.12 ± 1.22^e^	69.78 ± 1.98^d^	73.02 ± 3.65^d^	79.99 ± 2.09^c^	71.12 ± 2.56^c^	81.42 ± 1.23^b^	87.89 ± 2.69^a^

a The data are shown as the means of the reduction in biofilm generation (percentage) ± standard deviation (% ± SD). Differences in the superscript letters (a, b, c, d, e, f, g, h, j, and i) are statistically significant at *p* ≤ 0.05. R-sq (97.28%), adj R-sq (97.05%), and pred R-sq (96.76%).

For further examination of the time-kill kinetics of the T9 formula, pathogens with the strongest antimicrobial effects inside each microbial group were chosen. To find viable cells (%) following T9 treatment, the time-kill assay included strains of *Staphylococcus epidermidis* (gram +ve), *Salmonella paratyphi* (gram −ve), and *Candida albicans* (yeast cells). Table 4 summarizes the results for the time-kill kinetics data. The tested T9 formulation effectively decreases the number of viable planktonic cells for each tested pathogen. In all treated pathogens, the proportion of viable cells declined over the course of the culturing period in comparison to similar untreated cells (Fig. 9). The viable counts of planktonic cells were significantly reduced after 30 hours of incubation for *Staphylococcus epidermidis, Salmonella paratyphi*, and *Candida albicans* treated with the T9 formulation (Fig. 9i). *Staphylococcus epidermidis* (98.34 ± 2.37%), *Candida albicans* (97.28 ± 1.73%), and *Salmonella paratyphi* (94.09 ± 1.42%) showed the highest growth decrease percentages after pathogens were cultivated for 48 hours using the T9 formula (Fig. 9ii). These reduction percentages significantly increased following a 48 hour incubation period. Time-kill kinetics test was also used to calculate the duration of the T9 treatment that was essential to totally eradicate the pathogen biofilm. Whereas *Staphylococcus epidermidis* needed 54 hours to fully eradicate its biofilms, the treated biofilms of *Candida albicans* and *Salmonella paratyphi* were eliminated after 96 hours. Finally, the T9 combination consisting of 8 PMMA, 2 PVP, 2.5% drug A, and 2.5% drug B showed potential antimicrobial effects against microbial infection produced by multidrug-resistant human pathogens. To influence intracellular metabolic processes, the T9 formulation was designed to encourage the production of reactive oxygen species (ROS). These modifications make it viable for freshly produced ROS to destroy lipids, proteins, carbohydrates, and DNA.^3^

**Table 4:** *In vitro* time-kill kinetics of human infections treated with the T9 formula as well as the corresponding untreated cells. Calculations were conducted to determine the percentage of the biofilm that was reduced and the rate at which cell viability dropped throughout the incubation period^a^

Incubation period (h)	Multidrug-resistant human pathogens treated with the T9 formula (8 PMMA, 2 PVP, 2.5% drug A, and 2.5% drug B)								
	*Staphylococcus epidermidis*			*Salmonella paratyphi*			*Candida albicans*		
	Cell viability (log_10_ CFU ml^−1^ ± SD)		Biofilm reduction (% ± SD)	Cell viability (log_10_ CFU ml^−1^ ± SD)		Biofilm reduction (% ± SD)	Cell viability (log_10_CFU ml^−1^ ± SD)		Biofilm reduction (% ± SD)
	Untreated	Treated		Untreated	Treated		Untreated	Treated	
0	0.39 ± 0.06	0.33 ± 0.09	14.46 ± 0.56^e^	0.30 ± 0.37	0.29 ± 0.51	2.81 ± 0.37^e^	0.29 ± 0.53	0.29 ± 0.51	0.06 ± 0.16^e^
6	6.19 ± 0.60	3.24 ± 0.98	47.62 ± 2.69^de^	7.19 ± 1.28	5.24 ± 0.96	27.07 ± 2.94^de^	4.18 ± 0.96	3.64 ± 0.98	12.99 ± 2.84d^e^
12	6.24 ± 0.17	3.20 ± 0.51	51.95 ± 0.79^cd^	7.24 ± 0.68	4.51 ± 0.32	37.70 ± 2.21^cd^	5.41 ± 0.48	4.51 ± 1.32	16.65 ± 2.31^cd^
18	6.25 ± 0.48	2.27 ± 1.07	63.55 ± 1.81^bc^	7.24 ± 1.24	3.28 ± 0.75	54.73 ± 1.71^bc^	6.44 ± 0.28	2.28 ± 0.75	64.58 ± 3.12^bc^
24	6.26 ± 0.38	1.55 ± 0.67	75.11 ± 4.19^ab^	7.25 ± 0.98	2.75 ± 0.86	62.00 ± 3.18^ab^	7.36 ± 2.63	1.58 ± 0.73	78.46 ± 4.87^ab^
30	5.32 ± 0.25	1.45 ± 0.38	72.67 ± 3.68^ab^	6.31 ± 2.78	1.05 ± 0.38	83.31 ± 3.08^ab^	6.31 ± 0.64	1.08 ± 0.07	82.78 ± 2.89^ab^
36	4.32 ± 0.56	0.48 ± 0.54	88.75 ± 4.13^ab^	5.31 ± 0.78	0.78 ± 0.56	85.21 ± 1.84^ab^	6.31 ± 0.81	0.75 ± 0.64	88.01 ± 0.75^ab^
42	4.06 ± 0.19	0.25 ± 0.02	93.79 ± 2.52^a^	5.05 ± 0.81	0.55 ± 0.12	89.08 ± 0.23^a^	6.05 ± 0.42	0.59 ± 0.12	90.22 ± 3.23^a^
48	3.32 ± 0.02	0.05 ± 0.07	98.34 ± 2.37^a^	4.31 ± 0.78	0.25 ± 0.35	94.09 ± 1.42^a^	5.71 ± 0.46	0.15 ± 0.075	97.28 ± 1.73^a^

a SD = standard deviation. Differences in the superscript letters (a, b, c, d, and e) are statistically significant at *p* ≤ 0.05. R-sq (93.53%), adj R-sq (9066%), and pred R-sq (85.45%).

## 4. Conclusions

Nitrogen-bearing heterocyclic hybrid-loaded electrospun PMMA/PVP nanofibres were designed for the creation of new antimicrobial and anti-proliferative drug-loaded biomaterials. Our initial objective was to prepare a successful nitrogen-bearing heterocyclic hybrid PMMA/PVP blend to form the matrix, which was used as the main scaffold for the drugs, with anti-proliferative, antibacterial, and antifungal activities and greater selectivity toward the cancer cells, resulting in a higher selectivity index and lower IC_50_ than the reference drug. The antimicrobial activity of the nitrogen-bearing heterocyclic hybrid-PMMA/PVP blend to form the matrix was evaluated against different bacterial strains, as well as fungi, and displayed strong antimicrobial potency against all utilized resistant bacteria.

## Author contributions

Eman Abdelaziz and Asmaa M. Hasanein: experiments, data analysis and wrote the original draft; Tasneem Abed, Shahira H. EL-Moslamy and Mohamed A. M. Ali, Anis Ahmad Chaudhary, Fehmi Boufahja: experiments and microbiology part and cell culture, respectively. Samar A. Salim: data analysis, fabrication of nanofibers, formal analyses, and resources. Elbadawy A. Kamoun, Amr Negm, Mohamed A. Hawata and Ibrahim E.T. El Sayed: study design, supervision, wrote the original draft and reviewed the final draft. All authors have approved the manuscript before submission. All authors have critically reviewed and approved the final draft and are responsible for the content and similarity index of the manuscript.

## Conflicts of interest

The authors declare that they have no competing interests.

## Funding

This work was supported and funded by the Deanship of Scientific Research at Imam Mohammad Ibn Saud Islamic University (IMSIU) (grant number IMSIU-DDRSP2501).

## Supplementary Material

RA-015-D5RA02535D-s001

## Data Availability

All raw data of measurements is available and could be shared when requested, corresponding authors are fully responsible for providing all data requested. Supplementary information is available. See DOI: https://doi.org/10.1039/d5ra02535d.

## References

[cit1] Kirtane A. R., Verma M., Karandikar P., Furin J., Langer R., Traverso G. (2021). Nanotechnology approaches for global infectious diseases. Nat. Nanotechnol..

[cit2] Mallappa C. M., Choudhary N., Yadav K. K., Qasim M. T., Zairov R. (2025). *et al.*, Recent advances in the synthesis of nitrogen-containing heterocyclic compounds *via* multicomponent reaction and their emerging biological applications: a review. J. Iran. Chem. Soc..

[cit3] Abdelaziz E., El-Deeb N. M., Zayed M. F., Hasanein A. M., El Sayed I. E.-T., Elmongy E. I. (2023). *et al.*, Synthesis and in-vitro anti-proliferative with antimicrobial activity of new coumarin containing heterocycles hybrids. Sci. Rep..

[cit4] Mahlapuu M., Björn C., Ekblom J. (2020). Antimicrobial peptides as therapeutic agents: Opportunities and challenges. Crit. Rev. Biotechnol..

[cit5] Flores-Morales V., Villasana-Ruíz A. P., Garza-Veloz I., González-Delgado S., Martinez-Fierro M. L. (2023). Therapeutic effects of coumarins with different substitution patterns. Molecules.

[cit6] Alshibl H. M., Al-Abdullah E. S., Haiba M. E., Alkahtani H. M., Awad G. E., Mahmoud A. H. (2020). *et al.*, Synthesis and evaluation of new coumarin derivatives as antioxidant, antimicrobial, and anti-inflammatory agents. Molecules.

[cit7] Nuha D., Evren A. E., Kapusiz Ö., Gül Ü. D., Gundogdu-Karaburun N., Karaburun A. C. (2023). *et al.*, Design, synthesis, and antimicrobial activity of novel coumarin derivatives: An in-silico and in-vitro study. J. Mol. Struct..

[cit8] Tsivileva O. M., Koftin O. V., Evseeva N. V. (2022). Coumarins as fungal metabolites with potential medicinal properties. Antibiotics.

[cit9] Yildirim M., Poyraz S., Ersatir M. (2023). Recent advances on biologically active coumarin-based hybrid compounds. Med. Chem. Res..

[cit10] Husain A., Al Balushi K., Akhtar M. J., Khan S. A. (2021). Coumarin linked heterocyclic hybrids: a promising approach to develop multi target drugs for Alzheimer's disease. J. Mol. Struct..

[cit11] Reddy D. S., Kongot M., Kumar A. (2021). Coumarin hybrid derivatives as promising leads to treat tuberculosis: Recent developments and critical aspects of structural design to exhibit anti-tubercular activity. Tuberculosis.

[cit12] Zeydi M. M., Kalantarian S. J., Kazeminejad Z. (2020). Overview on developed synthesis procedures of coumarin heterocycles. J. Iran. Chem. Soc..

[cit13] Cheng F., Song D., Li H., Ravi S. K., Tan S. C. (2025). Recent progress in biomedical scaffold fabricated *via* electrospinning: design, fabrication and tissue engineering application. Adv. Funct. Mater..

[cit14] Mikayilov E., Tagiyev D., Zeynalov N., Tagiyev S. (2024). Exploring the Role of Poly (N-vinyl pyrrolidone) in Drug Delivery. Chem. Biochem. Eng. Q..

[cit15] Goh Y.-F., Shakir I., Hussain R. (2013). Electrospun fibers for tissue engineering, drug delivery, and wound dressing. J. Mater. Sci..

[cit16] Chen Y., Dong X., Shafiq M., Myles G., Radacsi N., Mo X. (2022). Recent advancements on three-dimensional electrospun nanofiber scaffolds for tissue engineering. Adv. Fiber Mater..

[cit17] Balavigneswaran C. K. , Ramesh K., Kaur R. and Misra N.. Versatility of Poly (Lactic Acid) and Modified Poly (Lactic Acid) for Nanobioengineering Applications. NanoBioEngineering: CRC Press; 2018. p. 145–166

[cit18] Kalangadan N., Mary A. S., Mani K., Nath B., Kondapalli J., Soni S. (2023). *et al.*, Repurposing ivermectin and ciprofloxacin in nanofibers for enhanced wound healing and infection control against MDR wound pathogens. J. Drug Delivery Sci. Technol..

[cit19] Ismail N. A., Salman A. A., Yusof M. S., Soh S. K., Ali H. M., Sarip R. (2018). The synthesis of a novel anticancer compound, N-(3, 5 Dimethoxyphenyl) acridin-9-amine and evaluation of its toxicity. Open Chem. Eng. J..

[cit20] Fatah S. M., Ahmed A. A., El-Bery E. N., Awad H. M., Abdel-Aleem A.-A. H., El-Sayed I. E.-T. (2025). *et al.*, Synthesis of bioactive benzopyridazine derivatives as antiproliferative agents against different cancer cell lines. Egypt. J. Chem..

[cit21] El-Gokha A., Shaban E., Mohamed A., Bahbah G. (2015). Synthesis and Antibacterial Activity of New Phthalazine Derivatives. Int. J. Res. Pharm. Sci..

[cit22] Mosmann T. (1983). Rapid colorimetric assay for cellular growth and survival: application to proliferation and cytotoxicity assays. J. Immunol. Methods.

[cit23] Abu-Serie M. M., El-Fakharany E. M. (2017). Efficiency of novel nanocombinations of bovine milk proteins (lactoperoxidase and lactoferrin) for combating different human cancer cell lines. Sci. Rep..

[cit24] Hudzicki J. (2009). Kirby-Bauer disk diffusion susceptibility test protocol. Am. Soc. Microbiol..

[cit25] Lewis I. and James S., Performance standards for antimicrobial susceptibility testing, Clinical and Laboratory Standards Institute, 33rd edn, 2023

[cit26] El-Moslamy S. H., Yahia I., Zahran H., Kamoun E. A. (2023). Novel biosynthesis of MnO NPs using Mycoendophyte: industrial bioprocessing strategies and scaling-up production with its evaluation as anti-phytopathogenic agents. Sci. Rep..

[cit27] El-Moslamy S. H., Abd-Elhamid A. I., Fawal G. E. (2024). Large-scale production of myco-fabricated ZnO/MnO nanocomposite using endophytic Colonstachys rosea with its antimicrobial efficacy against human pathogens. Sci. Rep..

[cit28] El-Moslamy S. H., Elnouby M. S., Rezk A. H., El-Fakharany E. M. (2023). Scaling-up strategies for controllable biosynthetic ZnO NPs using cell free-extract of endophytic Streptomyces albus: characterization, statistical optimization, and biomedical activities evaluation. Sci. Rep..

[cit29] Goher S. S., Aly S. H., Abu-Serie M. M., El-Moslamy S. H., Allam A. A., Diab N. H. (2024). *et al.*, Electrospun Tamarindus indica-loaded antimicrobial PMMA/cellulose acetate/PEO nanofibrous scaffolds for accelerated wound healing: In-vitro and in-vivo assessments. Int. J. Biol. Macromol..

[cit30] Abdelazim E. B., Abed T., Goher S. S., Alya S. H., El-Nashar H. A., El-Moslamy S. H. (2024). *et al.*, In vitro and in vivo studies of Syzygium cumini-loaded electrospun PLGA/PMMA/collagen nanofibers for accelerating topical wound healing. RSC Adv..

[cit31] EL-Moslamy S. H., Rezk A. H., Elkady M., Shokry Hassan H. (2024). Semi-industrial bio-fabrication of ZnO/MnO2 nanocomposite using endophytic streptomyces coelicolor: Characterization, statistical design, exponential pulse fed-batch fermentation, and its antimicrobial application. Arabian J. Sci. Eng..

[cit32] El-Bahnsawye M., Hussein M. K. A., Elmongy E. I., Awad H. M., Tolan A. A. E.-K., Moemen Y. S. (2022). *et al.*, Design, synthesis, and antiproliferative activity of novel neocryptolepine–rhodanine hybrids. Molecules.

[cit33] Laponogov I., Sohi M. K., Veselkov D. A., Pan X.-S., Sawhney R., Thompson A. W. (2009). *et al.*, Structural insight into the quinolone–DNA cleavage complex of type IIA topoisomerases. Nat. Struct. Mol. Biol..

[cit34] Koch A., Tamez P., Pezzuto J., Soejarto D. (2005). Evaluation of plants used for antimalarial treatment by the Maasai of Kenya. J. Ethnopharmacol..

[cit35] Le T. D., Pham N. N., Nguyen T. C. (2018). Preparation and Antibacterial Activity of Some New 4-(2-Heterylidenehydrazinyl)-7-chloroquinoline Derivatives. J. Chem..

[cit36] Elmongy E. I., Ahmed A. A., El Sayed I. E. T., Fathy G., Awad H. M., Salman A. U. (2022). *et al.*, Synthesis, Biocidal and Antibiofilm Activities of New Isatin–Quinoline Conjugates against Multidrug-Resistant Bacterial Pathogens along with Their In Silico Screening. Antibiotics.

[cit37] Marquez V. E., Cranston J. W., Ruddon R. W., Burckhalter J. H. (1974). Binding to deoxyribonucleic acid and inhibition of ribonucleic acid polymerase by analogs of chloroquine. J. Med. Chem..

[cit38] Abd Eldaim M. A., Tousson E., El S. I. E. T., Abd Elmaksoud A. Z., Ahmed A. A. (2021). Ameliorative effects of 9-diaminoacridine derivative against Ehrlich ascites carcinoma–induced hepatorenal injury in mice. Environ. Sci. Pollut. Res..

[cit39] Sharman W. M. , Van lier J. E.. Synthesis of Phthalocyanine 97. The Porphyrin Handbook: Phthalocyanines: Spectroscopic and Electrochemical Characterization. 2000;16:1

[cit40] Elbadry A. M., Kamoun E. A., Elsabahy M., Helmy M. S., EL-Moslamy S. H., Eissa N. G. (2025). *et al.*, Enhancing topical delivery of N-acetylcysteine and collagen via a novel electrospun collagen/PMMA nanofibrous mats as facial mask development: Nanofibers optimization and In vitro experiments. J. Drug Delivery Sci. Technol..

[cit41] Zhou J., Gao Q., Fukawa T., Shirai H., Kimura M. (2011). Macroporous conductive polymer films fabricated by electrospun nanofiber templates andtheir electromechanical properties. Nanotechnology.

[cit42] Mireles L. K., Wu M.-R., Saadeh N., Yahia L. H., Sacher E. (2020). Physicochemical characterization of polyvinyl pyrrolidone: A tale of two polyvinyl pyrrolidones. ACS Omega.

[cit43] Devikala S., Ajith D., Kamaraj P., Arthanareeswari M. (2019). Structural morphological and electrochemical studies on PMMA/PVP blends. Mater. Today: Proc..

[cit44] Russell L., Pène F., Martin-Loeches I. (2023). Multidrug-resistant bacteria in the grey shades of immunosuppression. Intensive Care Med..

[cit45] Saliba R., Zahar J.-R., Dabar G., Riachy M., Karam-Sarkis D., Husni R. (2023). Limiting the spread of Multidrug-resistant Bacteria in Low-to-Middle-Income countries: one size does not fit all. Pathogens.

[cit46] Dawoud N. T., El-Fakharany E. M., Abdallah A. E., El-Gendi H., Lotfy D. R. (2022). Synthesis, and docking studies of novel heterocycles incorporating the indazolylthiazole moiety as antimicrobial and anticancer agents. Sci. Rep..

[cit47] Mutagonda R. F., Marealle A. I., Nkinda L., Kibwana U., Maganda B. A., Njiro B. J. (2022). *et al.*, Determinants of misuse of antibiotics among parents of children attending clinics in regional referral hospitals in Tanzania. Sci. Rep..

[cit48] Breijyeh Z., Jubeh B., Karaman R. (2020). Resistance of gram-negative bacteria to current antibacterial agents and approaches to resolve it. Molecules.

[cit49] Feng X., Jin S., Wang M., Pang Q., Liu C., Liu R. (2020). *et al.*, The critical role of tryptophan in the antimicrobial activity and cell toxicity of the duck antimicrobial peptide DCATH. Front. Microbiol..

