# Supplementary material for: Coumarin/nitrogen-bearing heterocyclic hybrid-loaded electrospun PMMA/PVP nanofibrous scaffolds for accelerating topical wound healing rates: synthesis and in vitro bio-evaluation
Source: RSC Adv. 2025 Oct 3;15(44):36731–48. doi: 10.1039/d5ra02535d (PMC12495412; doi:10.1039/d5ra02535d)
Supplement: RA-015-D5RA02535D-s001 [file RA-015-D5RA02535D-s001.rtf]

Supplementary Information
Coumarin/Nitrogen-bearing heterocyclic hybrids loaded electrospun PMMA/PVP nanofibrous scaffolds for accelerating topical wound healing rate: Synthesis and in vitro bio-evaluation
Samar A. Salim1, Mohamed A. M. Ali2, Tasneem Abed3, Anis Ahmad Chaudhary2, Fehmi Boufahja2, Asmaa Mohammed Hasanein4, Eman Abdelaziz4, Shahira H. EL-Moslamy5, Amr Negm6*, Ibrahim E.T.  El Sayed4, Elbadawy A. Kamoun6*, Mohamed A. Hawata4

1Nanotechnology Research Center (NTRC), The British University in Egypt, El-Sherouk City, Cairo 11837, Egypt.
2Department of Biology, College of Science, Imam Mohammad Ibn Saud Islamic University (IMSIU), Riyadh 11623, Saudi Arabia.
3Badr University in Cairo Research Center, Badr University in Cairo, Badr City, Cairo 11829, Egypt.
4Department of Chemistry, Faculty of Science, Menoufia University, Shibin El Kom 32511, Egypt
5Bioprocess Development Department (BID), Genetic Engineering and Biotechnology Research Institute (GEBRI), City of Scientific Research and Technological Applications (SRTA-City), New Borg El-Arab City, Alexandria 21934, Egypt.
6Department of Chemistry, College of Science, King Faisal University, Al-Ahsa 31982, Saudi Arabia.

*Corresponding authors: Amr Negm, E-mail: anegm@kfu.edu.sa; E.A. Kamoun; E-mails: ekamoun@kfu.edu.sa and badawykamoun@yahoo.com, Tel: +201283320302, I. E.T. El-Sayed, E-mail: ibrahimtantawy@yahoo.co.uk.   
   

1.	Synthesis of 9-chloroacridine. 
9-Chloroacridine synthesized by modified Ullman-Goldberg reaction20. 
Modified Ullmann-Goldberg involves reaction of o-chlorobenzoic acid A  with aniline B in the presence of anhydrous K2CO3 , copper (acts as catalyst) and copper oxide (acts as co-catalyst) in DMF as solvent under reflux overnight at 136°C to give the acid intermediate C. Cyclization of the intermediate product C with phosphorus oxychloride under nitrogen atmosphere gives 9-chloroacridine 8 as depicted in Scheme 5. 
Yield (0.4gm, 80%), pale green solid, m.p. 108-111°C (lit. 116°C).  IR (KBr) cm-1: 3073 (C-H, Ar.), 1626 (C=N), 1547 (C=C, Ar.), 1271 (C-N). ¹H -NMR (CDCl3, 300 MHz), ä (ppm): 7.66-7.71 (m, 2H, Ar-H), 7.84-7.89 (m, 2H, Ar-H), 8.31-8.34 (d, J = 8.4 Hz, 2H, Ar-H), 8.46-8.49 (d, J =8.7Hz, 2H, Ar-H).

Scheme 5: Synthesis  of 9-chloroacridine.

2.	Synthesis of 1-chloro-4-(p-tolyl) phthalazine:
To a mixture  of  phthalic  anhydride D (0.01 mol) and  (25 mL, 0.2 mol) of toluene,   (0.015 mol), AlCl3 anhydrous  was  added  gradually  and reaction mixture was heated  for  2h  on water bath, and poured onto ice/dilute HCl. The formed semisolid product was treated with petroleum ether, and the obtained solid was filtered off to afford the pure acid intermediate E.  A solution of acid E (0.01 mol) in absolute ethanol (15 mL), hydrazine hydrate (0.01 mol) was added, and the reaction mixture was refluxed for 4 h. After cooling, the precipitated solid was filtered off and crystallized from ethanol to afford the intermediate phthalazinone F.  A mixture of phthalazinone F (0.01 mol), and phosphorus oxychloride (3 mL) was refluxed for 4 h on a steam bath. After cooling, the reaction mixture was poured carefully onto crushed ice. The separated solid was filtered off,washed well for several times with water, dried, and crystallized from ethanol to afford the key intermediate 11 as displayed in Scheme 6. Yield (1.14 g, 79%), brown crystals, m.p: 148-150 °C; IR(KBr): IR spectrum (KBr, í cm-1): 1662 (C═N), 835 (C–Cl); 759 (C-Cl); 1HNMR (400 MHz, DMSO-d6) ppm: 2.44 (s, 1H, Ar-CH3), 7.41 (d, 2H, J = 8.4 Hz), 7.6 (d, 2H, J = 8.1 Hz), 8.02 (m, 3H), 8.36 (d, 1H, J = 7.8Hz).
 
                                            Scheme 6: Synthesis  of 1-chloro-4-(p-tolyl) phthalazine.

Table 1S: List of different formulations and spinning parameters optimization for fabrication of electrospun PMMA/PVP nanofiber scaffolds.
Formulation	Voltage (Kv)	Feed rate (mL h−1)	Observation	
8PMMA: 2PVP/ 0% drug	24	0.6	Fiber with no beads.	
8PMMA: 2PVP/ 0.5% drug (A)	24	1.0	Good fiber with no beads.	
8PMMA: 2PVP/ 1%  drug (A)	24	0.9	Good fiber with no beads.	
8PMMA: 2PVP/ 3%  drug (A)	24	0.6	Good fiber with no beads.	
8PMMA: 2PVP/ 5%  drug (A)	22	0.7	Good fiber with no beads.	
8PMMA: 2PVP/ 0.5% drug (B)	23	0.9	Good fiber with no beads.	
8PMMA: 2PVP/ 1%    drug (B)	23	0.9	Good fiber with no beads.	
8PMMA: 2PVP/ 3%    drug (B)	23	0.6	Good fiber with no beads.
Dilated diameter.	
8PMMA: 2PVP/ 5%    drug (B)	27	0.5	Good fiber with no beads.
Dilated diameter.	
8PMMA: 2PVP/ 2.5% drug (A)/ 2.5% drug (B)	24	0.7	Good fiber with no beads.
Dilated diameter.	
             Note: drug A: Coumarin-quinoline and drug B: Coumarin-acridine.
